# Independent Maternal and Fetal Genetic Effects on Midgestational Circulating Levels of Environmental Pollutants

**DOI:** 10.1534/g3.117.039784

**Published:** 2017-02-24

**Authors:** Michela Traglia, Lisa A. Croen, Kristen Lyall, Gayle C. Windham, Marty Kharrazi, Gerald N. DeLorenze, Anthony R. Torres, Lauren A. Weiss

**Affiliations:** * Department of Psychiatry, Weill Institute for Neurosciences and Institute for Human Genetics, University of California, San Francisco, California 94143; † Autism Research Program, Division of Research, Kaiser Permanente, Oakland, California 94612; ‡ Division of Environmental and Occupational Disease Control, California Department of Public Health, Richmond, California 94804; § Genetic Disease Screening Program, California Department of Public Health, Richmond, California 94804; ** Center for Persons with Disabilities, Utah State University, Logan, Utah 84322

**Keywords:** environmental pollutants, autism spectrum disorders, organohalogens, heritability, GWAS, metabolism

## Abstract

Maternal exposure to environmental pollutants could affect fetal brain development and increase autism spectrum disorder (ASD) risk in conjunction with differential genetic susceptibility. Organohalogen congeners measured in maternal midpregnancy blood samples have recently shown significant, but negative associations with offspring ASD outcome. We report the first large-scale maternal and fetal genetic study of the midpregnancy serum levels of a set of 21 organohalogens in a subset of 790 genotyped women and 764 children collected in California by the Early Markers for Autism (EMA) Project. Levels of PCB (polychlorinated biphenyl) and PBDE (polybrominated diphenyl ether) congeners showed high maternal and fetal estimated SNP-based heritability (*h*^2^*_g_*) accounting for 39–99% of the total variance. Genome-wide association analyses identified significant maternal loci for p,p′-DDE (*P* = 7.8 × 10^−11^) in the *CYP2B6* gene and for BDE-28 (*P* = 3.2 × 10^−8^) near the *SH3GL2* gene, both involved in xenobiotic and lipid metabolism. Fetal genetic loci contributed to the levels of BDE-100 (*P* = 4.6 × 10^−8^) and PCB187 (*P* = 2.8 × 10^−8^), near the potential metabolic genes *LOXHD1* and *PTPRD*, previously implicated in neurodevelopment. Negative associations were observed for BDE-100, BDE153, and the sum of PBDEs with ASD, partly explained by genome-wide additive genetic effects that predicted PBDE levels. Our results support genetic control of midgestational biomarkers for environmental exposures by nonoverlapping maternal and fetal genetic determinants, suggesting that future studies of environmental risk factors should take genetic variation into consideration. The independent influence of fetal genetics supports previous hypotheses that fetal genotypes expressed in placenta can influence maternal physiology and the transplacental transfer of organohalogens.

Autism spectrum disorders (ASD) are complex disorders that affect brain development, and are characterized by repetitive behaviors, deficits in use of language and social communication, sensory dysfunction, and restricted interests that emerge during infancy. Available genetic data based on family studies and twin studies have highlighted high heritability for ASD between 40 and 90% (Gaugler *et al.* 2014; Sandin *et al.* 2014) and a strong polygenic contribution, but single major genetic risk factors are identified in at best 20% of cases (Abrahams and Geschwind 2008). Recent studies have focused on the identification of nongenetic determinants of ASD risk and suggested a strong contribution of environmental factors (Grandjean and Landrigan 2014; Rice and Barone 2000) and of immune system dysregulation (Bilbo and Schwarz 2012; Goines *et al.* 2011; Mead and Ashwood 2015). These findings, combined with strong evidence of ASD’s prenatal origins (Bauman and Kemper 2005), suggest that fetal development and ASD risk might be influenced by a complex interplay between genetic and environmental risk factors.

Several organohalogen compounds, such as persistent organochlorine pesticides (OCPs), polychlorinated biphenyls (PCBs), and flame retardants like polybrominated biphenyls (PBBs) and polybrominated diphenyl ethers (PBDEs) widely used in industrial and domestic settings in the past, have been phased out from use in the United States, but due to their persistence continue to be detected in the bloodstream, liver, breast milk, adipose tissue, and placenta (Leonetti *et al.* 2016). Several studies in animal models provide evidence that exposure to PCBs and PBDEs during development can adversely affect behavior (Korrick and Sagiv 2008; Lilienthal *et al.* 2006), learning and memory (Eriksson *et al.* 2001), thyroid function (Legler and Brouwer 2003; McDonald 2002), and dopaminergic neurotransmission (Choksi *et al.* 1997). Furthermore, exposure to p,p′-DDE has been found to adversely affect the immune system (Rogan and Chen 2005) and glucose metabolism (Debost-Legrand *et al.* 2016). During pregnancy, organohalogens that have accumulated in the maternal bloodstream cross the placenta and may disrupt fetal brain development and influence fetal immune system development or fetal endocrine function, which in turn may also contribute to the risk of ASD. Previous studies have reported conflicting associations for different PBDE and PCB congeners with ASD symptoms (Braun *et al.* 2014; Mitchell *et al.* 2012; Nowack *et al.* 2015). Recently, two papers from the Early Markers for Autism (EMA) study – a population-based case–control study of autism and developmental disability in Southern California – have shown significant association between several organohalogens measured in maternal serum midpregnancy and ASD outcome in offspring: PCB138/158 and PCB153 were positively associated with ASD outcome, but surprisingly, levels of BDE-47, -99, -100, -153, and the sum of these PBDE congeners were negatively associated with ASD outcome (Lyall *et al.* unpublished results, 2016). It is the objective of this paper to determine the role of unexplored factors such as genetic determinants of or interactions with PCB and PBDE levels on these observations.

Our primary hypothesis was that the circulating midgestational levels of organohalogens would be driven by common maternal genetic determinants, and that these results could shed light on the observed associations between the organohalogens and ASD. We carried out the first genome-wide maternal SNP-based heritability and association analyses of maternal serum levels of organohalogens and we assessed whether the genome-wide associated genetic factors were also associated with ASD outcome. Additionally, we hypothesized that fetal genetics might be associated with the midgestational maternal circulating levels of organohalogens, actively regulating the maternal–fetal chemical transfer and accumulation.

## Materials and Methods

### Study population and blood sampling

Our study population was derived from the EMA study (Croen *et al.* 2008; Tsang *et al.* 2013), a population-based nested case–control study that included mother–infant pairs drawn from the population of pregnant women (15–20 wk) in Orange, San Diego, and Imperial Counties, California, who were enrolled in the State’s Prenatal Expanded Alphafetoprotein Screening Program and delivered a liveborn infant in 2000–2003 with a newborn screening bloodspot available. The offspring outcome of ASD was ascertained from the client files of the two regional centers (RCs) providing services to individuals with developmental disabilities living in these counties and verified by study clinician expert review of records according to a protocol developed by the Metropolitan Atlanta Developmental Disabilities Surveillance Program, as described previously (Tsang *et al.* 2013). The controls were randomly sampled from the birth certificate files and matched to ASD cases by sex, birth month, and birth year (Tsang *et al.* 2013). Maternal blood samples were collected in serum separator tubes at 15–20 wk gestation and stored as part of Project Baby’s Breath. Serum was stored in cryovials and cell pellets stored in Serum separator tubes (SSTs) at −20°. Newborn blood spots were collected on filter paper 1–2 d after birth and stored at −20° and maintained by the Genetic Disease Screening Program, California Department of Public Health. Six polybrominated congeners (BB-153, BDE-28, -47, -99, -100, -153), 11 polychlorinated congeners (PCB28, 99, 118, 138/158, 153, 170, 180, 187, 194, 196/203, 199), and two OCPs [p,p′-dichlorodiphenyldichloroethene (p,p′-DDE) and *trans*-nonachlor (T-NONA)] were measured in maternal serum by gas chromatography isotope dilution high-resolution mass spectrometry as previously described in Sjödin *et al.* (2004). Concentrations of these organohalogens were reported as *ng/g* lipid weight subtracted for the analytical background detected in blank samples. All concentration data were corrected for the average amount present in blank samples, and three blanks were included in every set of 30 samples as previously described (Sjödin *et al.* 2004). All study procedures were approved by the institutional review boards of the California Health and Human Services Agency and Kaiser Permanente Northern California; it was also determined by the University of California, San Francisco (UCSF) Committee on Human Research that the institution was not engaged in research on human subjects.

### DNA extraction and genotyping

The QIAGEN QIAamp 96 DNA Blood Kit was used to extract DNA from a subset of maternal and neonatal blood samples and the Invitrogen Quant-iT DNA Assay Kit to measure the DNA concentration by the biomedical laboratory at Utah State University, as previously described (Tsang *et al.* 2013). For newborn samples, the dried bloodspot (about 14 mm in diameter) collected on filter paper was entirely punched with a 3.2-mm paper punch. Since there is little DNA in a 3.2-mm paper punch, 15–18 punches were used for DNA extraction. Maternal and neonatal samples were genotyped using the Affymetrix Axiom (Affymetrix 2011) EUR array (675,000 SNPs across the genome) by the Genomics Core Facility (GCF) at UCSF, using standard protocols. Genotype calling was carried out using Affymetrix Power Tools (“Affymetrix Power Tools, Affymetrix Website”), as previously described (Tsang *et al.* 2013).

Individual-based and marker-based quality controls were performed with PLINK software (Purcell *et al.* 2007; http://zzz.bwh.harvard.edu/plink/) as reported in Tsang *et al.* (2013). We evaluated Mendelian errors of inheritance in all of our complete maternal–neonatal pairs and excluded markers with ≥0.10 of these errors. We also excluded markers that violated Hardy–Weinberg Equilibrium (HWE) in control mothers (*P*-value < 10^−10^) using the Hardy–Weinberg exact test statistic. Additionally, we extracted only common SNPs (MAF ≥ 1%). Two high-quality datasets were used in our analysis: the first dataset included 790 maternal samples (390 ASD cases, 400 controls) and 629,686 genotyped markers and the second dataset included 764 neonatal samples (385 ASD cases, 379 controls) and 622,716 genotyped markers. Most of these were related pairs of maternal–neonatal samples (366 case pairs, 369 control pairs). The maternal dataset was a subset of those with chemical biomarker levels previously reported (Lyall *et al.* 2015, 2016) (1144 maternal samples: 545 with offspring affected with ASD, 181 with intellectual disability, and 418 controls).

### Ancestry analysis

The distribution of maternal race/ethnicity as documented on the birth certificate was as follows: 42% Hispanic, 35% non-Hispanic Caucasian, 15% Asian, 3% South Asian, and 3% African American (Supplemental Material, Table S1 in File S1). We also carried out genome-wide multi-dimensional scaling (MDS) analysis separately on high-quality markers genotyped in women and in newborns included in our dataset using a pairwise distance genomic matrix and the multi-dimensional scale functions implemented in PLINK software (Purcell *et al.* 2007) (–cluster and–mds plot). The resulting maternal and fetal genetic matrices included 10 principal coordinates that summarized the genetic distance between each maternal and neonatal sample. The proportion of the total genetic variance accounted by the first two MDS principal coordinates (PC1, PC2) were estimated by princomp() command in the R 3.2.0 environment (R Core Team 2014) and plotted for maternal and neonatal genotypes (Figure S1, a and b in File S1). The genetic distances among different ancestry groups were highlighted in the PC1 and PC2 chart: most of the women were contained within a distinct ancestry cluster matching their self-reported group (Table S1 in File S1), but 10% of them were between clusters (Figure S1c in File S1). In order to account for population stratification, we included the first 10 principal coordinates in our analysis. In addition, we calculated a two-sample Mann–Whitney Wilcoxon test in R 3.2.0 (R Core Team 2014) environment to test whether, within each ancestry cluster, cases and controls showed significant differences for each MDS principal coordinate: Hispanic women showed significant differences between cases and controls along the first two principal coordinates (Table S1 in File S1). Non-Hispanic clusters include well-matched proportions of mothers of ASD cases and mothers of controls. Hispanic controls exceed the number of cases, as expected based on lower ASD rates in Hispanic families reported in the literature (Windham *et al.* 2011; Croen *et al.* 2002; Autism and Developmental Disabilities Monitoring Network Surveillance 2007).

### Confounding factors for PBB, PBDE, and PCB serum levels

The log-transformed serum levels of BB-153, two OCPs (T-NONA and p,p′-DDE), five PBDEs (BDE-28, -47, -99, -100, -153), and 11 PCB congeners (28, 99, 118, 138/158, 153, 170, 180, 187, 194, 196/203, 199) for which at least 60% of the study group had values above the limit of detection (LOD), except BDE-28 (only detected in 55% of the study group), were included in this study. Two additional variables for total PBDEs and PCBs were created by summing the individual concentrations of congeners with detection rates >60% (Sum PBDE and Sum PCB), as previously described (Lyall *et al.* 2015, 2016). We also examined the sum of congeners with detection rates >80% but we did not find any differences compared with the less stringent criteria. For participants with measurements below the LOD, values were replaced with LOD/√2 (Sjödin *et al.* 2013; Axelrad *et al.* 2009).

After outlier exclusion, using a threshold of 3 or 4 SD from the mean (based on normality tests of organohalogen distributions), we analyzed the effect of available sociodemographic covariates on the levels of BB-153, OCPs, PBDEs, and PCBs with linear regression models in the subset of genotyped women using the R 3.2.0 environment (R Core Team 2014) (Table S2 in File S1). Following Lyall *et al.* (2015) we included several confounding factors, such as maternal country of birth (USA, Mexico, other) and age at midpregnancy (15–45 yr old) and offspring gender and month of birth, as well as offspring ASD affection status, maternal educational attainment (elementary, high school, college, postgraduate), and genetic ancestry (the first 10 coordinates).

We tested the correlation between organohalogens and the first 10 ancestry principal coordinates in three subsets of women selected from distinct ancestry groups. Hispanic (*N* = 284), Caucasian (*N* = 261), and Asian (*N* = 116) women were separately analyzed (Figure S1c in File S1). After ancestry adjustment, the mean value of the residual levels of congeners did not show significant differences among ancestry groups (two-sample Mann–Whitney Wilcoxon test *P* > 0.1), suggesting that the first 10 principal coordinates accounted for bias due to population stratification (data not shown).

### Offspring ASD outcome association with congeners

After adjustment for the sociodemographic covariates listed above via regression, we tested for differences between the residuals for BB-153, OCPs, PBDEs, and PCBs in mothers of ASD cases and mothers of controls with a two-sample Mann–Whitney Wilcoxon test in the R 3.2.0 environment (R Core Team 2014). We assessed the linear correlation across congeners with Spearman’s test implemented in R 3.2.0 “corrplot” package (R Core Team 2014).

### SNP-based heritability and genetic correlation

The final set of maternal and neonatal autosomal high-quality markers were used to generate genetic-relationship matrices and calculate marker-based additive heritability (*h*^2^*_g_*) of organohalogen serum levels with a Restricted Maximum Likelihood [REML (Yang *et al.* 2010)] model implemented in GCTA software (Yang *et al.* 2011; http://cnsgenomics.com/software/gcta/). The heritability estimation indicates the proportion of the total organohalogen levels (phenotypic variance or V_p_) accounted for by the genetic variance (V_g_/V_p_) after the exclusion of the effect of the sociodemographic and case/control status covariates. We assessed the power of the heritability estimation in our dataset via the REML approach with GCTA-GREML Power Calculator (Visscher *et al.* 2014). To determine whether correlated organohalogens (see above) or related organohalogens (Lyall *et al.* 2015, 2016) shared genetic determinants, we estimated the genetic correlations (*r_g_*) between all possible organohalogen pairs using the estimated heritability (*h*^2^*_g_*) for each compound in a bivariate REML (Lee *et al.* 2012) model in GCTA software (Yang *et al.* 2011) and compared them to the linear correlations across each pair with cor.test() in the R 3.2.0 “stats” package (R Core Team 2014). We applied Fisher’s z transformation test in the R 3.2.0 environment (R Core Team 2014) to assess significant differences between estimated correlation coefficients. To assess whether the genetic factors that explain organohalogen levels (via SNP-based heritability) might also explain the reported association between organohalogen levels and ASD outcome, we estimated organohalogen-specific best linear unbiased prediction (BLUP) values for each individual adjusted for the sociodemographic covariates with the GCTA software (Yang *et al.* 2011). Individual BLUP values summarize, for a specific organohalogen, the individual total genetic effects explained by the genome-wide SNPs excluding the effect of the confounding factors. We then included BLUP variables in organohalogen-specific linear regression models as covariates. We used the obtained organohalogen residuals after adjustment for BLUP in linear regression models with ASD outcome variable to assess the association between ASD outcome and the “nongenetic” variation in organohalogen levels.

### Genome-wide association study

We performed congener-specific genome-wide association studies (GWAS) for maternal and fetal common SNPs using a linear model implemented in PLINK software (Purcell *et al.* 2007) (–linear, taking into account the effect of the covariates). Then, we used the Locuszoom (Pruim *et al.* 2011; http://locuszoom.sph.umich.edu/locuszoom/) tool to generate regional genomic plots and assess linkage disequilibrium (LD) among associated SNPs. We use the genome-wide significance threshold (*P* < 5 × 10^−8^) and suggestive threshold (*P* < 1 × 10^−7^) to account for approximate independent common polymorphism testing per GWAS (Risch and Merikangas 1996; Pe’er *et al.* 2008). We have tested many correlated biomarkers and performed testing in related mothers and offspring; although these add to the multiple testing burden, they are nonindependent, thus we were unable to calculate exact correction for study-wide significance and present uncorrected *P*-values.

### Maternal and fetal contribution to the associated loci

For each maternal organohalogen that showed a genome-wide significant maternal locus that is also suggestively associated in fetal genetics, we assessed whether the association were controlled by maternal and/or fetal genetics. We performed linear regression modeling including the whole set of covariates and the genotypes of maternal- and fetal-associated SNPs, and we assessed the residual association for maternal and fetal genotypes.

### Genetic determinants affecting organohalogen–ASD association

We wanted to understand whether genetic determinants associated with pollutants demonstrating statistically significant associations with ASD might drive the association between organohalogens and ASD or show interaction effects. Thus, we selected each maternal and neonatal compound-specific top SNP for pollutants associated with ASD and we included each genotype in distinct linear models for log-transformed organohalogen levels and in logistic regression models for ASD outcome, and we assessed both main effects and interaction terms with affection status. We then assessed whether the top SNPs changed the association between log-transformed organohalogen levels and ASD or whether the top SNPs interact with ASD. We calculated with mRnd (Brion *et al.* 2013) whether our dataset had enough power for a Mendelian Randomization analysis (Elliott *et al.* 2009), an approach that takes advantage of the random allocation of the alleles at conception to demonstrate that a genetic variant known to modify the level of a quantitative trait (*e.g.*, PBDE) could also modify the disease risk (*e.g.*, ASD) and may represent indirect evidence of a causal association between the quantitative trait and disease.

### Data availability

The full genome-wide summary-level results for each congener analyzed are available in two compressed supplemental material tables (Table S3 and Table S4).

## Results

### Midgestational organohalogen associations with offspring ASD outcome

We examined the association of midgestational concentrations of 19 organohalogens and the sums of PBDEs and PCBs (Sum PBDE, Sum PCB) with ASD outcome in the genotyped subset of the EMA cohort used for this study. We replicated significant or suggestive negative association for three polybrominated compounds (BDE-100, 153, and Sum PBDE *P* < 0.05) (Figure S2 in File S1 and Table 1) (Lyall *et al.* 2016). No significant association with the persistent pesticides or OCPs (Lyall *et al.* 2015) (p,p′-DDE and T-NONA) was detected; nor did we observe the positive nominally significant association between PCBs and ASD outcome (Lyall *et al.* 2015) in our analyses parameterizing exposure continuously (Table 1). However, when analyzing PCB138/158 or 153 using a quartile analysis approach (Table S5 in File S1) we found consistent results with the previous findings shown in the full EMA sample (Lyall *et al.* 2015).

**Table 1:** ASD outcome association with organohalogens in mothers after adjustment for sociodemographic covariates and for maternal and fetal ancestry

	Maternal Ancestry-Adjusted					Fetal Ancestry-Adjusted				
Compound	*N*	β	SE	OR (95% CI)	*P*-Value**^a^**	*N*	β	SE	OR (95% CI)	*P*-Value**^a^**
Persistent pesticides										
p-p′-DDE	762	−0.08	0.06	0.92 (0.82–1.04)	NS	708	−0.08	0.07	0.92 (0.80–1.06)	NS
T-NONA	706	0.01	0.04	1.01 (0.94–1.09)	NS	656	−0.01	0.04	0.99 (0.92–1.07)	NS
Polybrominated diphenyl ethers and biphenyls										
BB-153	705	−0.01	0.05	0.99 (0.90–1.09)	NS	655	−0.02	0.05	0.98 (0.89–1.08)	NS
BDE-28	698	−0.03	0.05	0.97 (0.88–1.07)	NS	649	−0.02	0.05	0.98 (0.89–1.08)	NS
BDE-47	762	−0.14	0.08	0.87 (0.74–1.02)	0.07	708	−0.13	0.08	0.88 (0.75–1.03)	NS
BDE-99	741	−0.13	0.07	0.88 (0.77–1.01)	0.07	688	−0.13	0.08	0.88 (0.75–1.03)	0.09
BDE-100	750	−0.18	0.08	0.84 (0.71–0.98)	1.97 × 10^−2^	697	−0.2	0.08	0.82 (0.70–0.96)	1.4 × 10^−2^
BDE-153	749	−0.2	0.07	0.82 (0.71–0.94)	6.54 × 10^−3^	695	−0.22	0.08	0.82 (0.69–0.94)	4.1 × 10^−3^
Sum PBDE	710	−0.16	0.07	0.85 (0.74–0.98)	1.74 × 10^−2^	659	−0.18	0.07	0.84 (0.73–0.96)	1.1 × 10^−2^
Polychlorinated biphenyls										
PCB28	763	0.13	0.09	1.39 (0.96–1.26)	NS	709	0.12	0.09	1.1 (0.95–1.35)	NS
PCB99	712	−0.01	0.04	0.99 (0.92–1.07)	NS	662	−0.02	0.04	0.98 (0.91–1.06)	NS
PCB118	714	0.01	0.04	0.99 (0.92–1.07)	NS	664	−0.01	0.04	0.99 (0.92–1.07)	NS
PCB153	755	0.01	0.04	0.99 (0.92–1.07)	NS	703	−0.01	0.04	0.99 (0.92–1.07)	NS
PCB170	727	−0.01	0.04	0.99 (0.92–1.07)	NS	675	−0.03	0.04	0.97 (0.90–1.05)	NS
PCB180	759	0.01	0.04	0.99 (0.92–1.07)	NS	705	−0.01	0.04	0.99 (0.92–1.07)	NS
PCB187	714	−0.01	0.04	0.99 (0.92–1.07)	NS	664	−0.02	0.05	0.98 (0.89–1.08)	NS
PCB194	712	−0.02	0.04	0.98 (0.91–1.06)	NS	662	−0.03	0.04	0.97 (0.89–1.05)	NS
PCB199	715	−0.02	0.05	0.97 (0.89–1.05)	NS	665	−0.03	0.05	0.97 (0.88–1.07)	NS
PCB138/158	752	0.01	0.04	0.99 (0.92–1.07)	NS	698	−0.01	0.05	0.99 (0.90–1.09)	NS
PCB196/203	715	−0.05	0.04	0.95 (0.88–1.03)	NS	665	−0.04	0.04	0.97 (0.89–1.04)	NS
Sum PCB	706	0.03	0.05	1.03 (0.93–1.13)	NS	657	0.01	0.05	1.01 (0.92–1.11)	NS

SE, standard error; OR, odds ratio; CI 95%, confidence interval 95%.

a Significance of ASD outcome in each congener linear regression model.

### Maternal SNP-based heritability of midgestational organohalogen levels

Maternal common genetic factors accounted for 39–93% of the variance in organohalogen levels across the set of polybrominated compounds, but they showed high SEs and estimates were nonsignificant for BDE-28 and BDE-99 (Table 2). There was no evidence of heritability for OCPs, whereas we found some degree of heritability, although not statistically significant, for a few PCB congeners (Table 2). Our modest sample size did not reach sufficient power for a reliable estimation of SNP-based heritability (power = 70% to estimate *h*^2^*_g_* = 0.99), thus lack of significance may not indicate absence of heritability.

**Table 2:** SNP-based maternal and fetal heritability for polybrominated diphenyl ethers (PBDEs) and 2,2′,4,4′,5,5′-hexabromobiphenyl (BB-153) in maternal serum

	Maternal Genetics			Fetal Genetics		
Compound	*h*^2^*_g_*	SE	*P*-Value	*h*^2^*_g_*	SE	*P*-Value
Persistent pesticides						
p,p′-DDE	0	0.32	NS	0	0.36	NS
T-NONA	0	0.35	NS	0	0.37	NS
Polybrominated diphenyl ethers and biphenyls						
BB-153	0.93	0.31	4.5 × 10^−3^	0.98	0.33	4.0 × 10^−3^
BDE-28	0.53	0.33	NS	0.82	0.34	1.3 × 10^−2^
BDE-47	0.73	0.31	1.6 × 10^−2^	0.99	0.31	1.6 × 10^−3^
BDE-99	0.39	0.33	NS	0.66	0.34	2.3 × 10^−2^
BDE-100	0.93	0.29	1.7 × 10^−3^	0.99	0.32	7.2 × 10^−5^
BDE-153	0.79	0.3	5.4 × 10^−3^	0.99	0.31	4.7 × 10^−4^
Sum PBDE	0.77	0.31	8.3 × 10^−3^	0.91	0.33	5.2 × 10^−3^
Polychlorinated biphenyls						
PCB28	0	0.32	NS	0	0.36	NS
PCB99	0	0.35	NS	0.06	0.37	NS
PCB118	0.07	0.34	NS	0	0.38	NS
PCB153	0.36	0.32	NS	0.58	0.34	4.54 × 10^−2^
PCB170	0.39	0.33	NS	0.37	0.36	NS
PCB180	0.15	0.32	NS	0.1	0.35	NS
PCB187	0.42	0.33	NS	0.47	0.36	NS
PCB194	0	0.34	NS	0	0.37	NS
PCB199	0.38	0.32	NS	0.17	0.36	NS
PCB138/158	0.44	0.31	NS	0.57	0.34	4.58 × 10^−2^
PCB196/203	0.12	0.33	NS	0	0.36	NS
Sum PCB	0	0.36	NS	0.09	0.38	NS

*h*^2^*_g_*, proportion of genetic variance on the total phenotypic variance; SE, standard error; NS, not significant.

We estimated linear correlations within and between different categories of organohalogens (Figure S3 in File S1). To assess patterns of organohalogens that share genetic determinants, we calculated the coheritability (genetic correlation) intra- and interclass between all organohalogen pairs, and we compared genetic correlations to linear correlations. We expected that genetic correlation between two chemicals might be higher than linear correlation if there are differences in the sources of exposure among organohalogens and/or a different impact of additional environmental factors, such as diet or lifestyle, that may contribute to the accumulation of organohalogens but similar genetic determinants relevant to multiple organohalogens. In contrast, significantly lower genetic correlations than linear correlations could indicate coexposure and/or similar contribution of contributing environmental factors but different genetic determinants. Most of the significant genetic correlation coefficients were also significantly higher than the matched linear correlation coefficients except for BDE-153 pairwise correlations with BDE-28, BDE-99, and BDE-100 (Table 3).

**Table 3:** Significant genetic correlation across covariate-adjusted organohalogen serum levels and corresponding linear correlation

		Maternal Genetics				Fetal Genetics			M/F *r_g_*
Compound	Compound	*N*	*r_g_* (SE)	*r_res_*	*r_g_* ≠ *r_res_ P**^a^*	*N*	*r_g_* (SE)	*P*-Value*^a^*	*Mr_g_* ≠ *Fr_g_ P**^b^*
BDE-28	BDE-100	698	0.92*** (0.08)	0.77***	<0.01	649	NC	ND	ND
BDE-28	BDE-153	697	0.34*** (0.29)	0.56***	2.1 × 10^−7^	648	0.66*** (0.21)	3.4 × 10^−3^	1.1 × 10^−15^
BDE-47	BDE-99	741	0.98*** (0.04)	0.93***	<0.01	688	NC	ND	ND
BDE-47	BDE-100	750	1.00*** (0.04)	0.93***	<0.01	697	NC	ND	ND
BDE-47	BDE-153	749	0.61* (0.18)	0.65***	NS	696	0.77*** (0.09)	3.4 × 10^−6^	3.6 × 10^−9^
BDE-99	BDE-100	741	0.99*** (0.07)	0.88***	<0.01	688	NC	ND	ND
BDE-99	BDE-153	740	0.34*** (0.31)	0.60***	9.7 × 10^−12^	687	0.66*** (0.22)	NS	2.2 × 10^−16^
BDE-100	BDE-153	749	0.64*** (0.16)	0.82***	1.4 × 10^−14^	696	NC	ND	ND
PCB180	PCB138/158	752	1.00*** (0.40)	0.81***	<0.01	698	NC	ND	ND
PCB180	PCB187	714	0.80*** (0.33)	0.86***	2.4 × 10^−4^	664	NC	ND	ND

*r_g_*, genetic correlation; SE, standard error; *r_res_*, significant Spearman’s linear correlation across residual congener levels after covariate adjustment; *P*, *P*-value; M/F, maternal/fetal; NS, not significant; NC, not converged; ND, not done. *** *P* < 0.001, ** *P* < 0.01, * *P* < 0.05.

a Fisher’s z transformation test between residual and genetic correlation coefficients.

b Fisher’s z transformation test between maternal genetic and fetal genetic correlation coefficients.

### Maternal genome-wide association analysis of midgestational organohalogen concentrations

**Figure 1:**
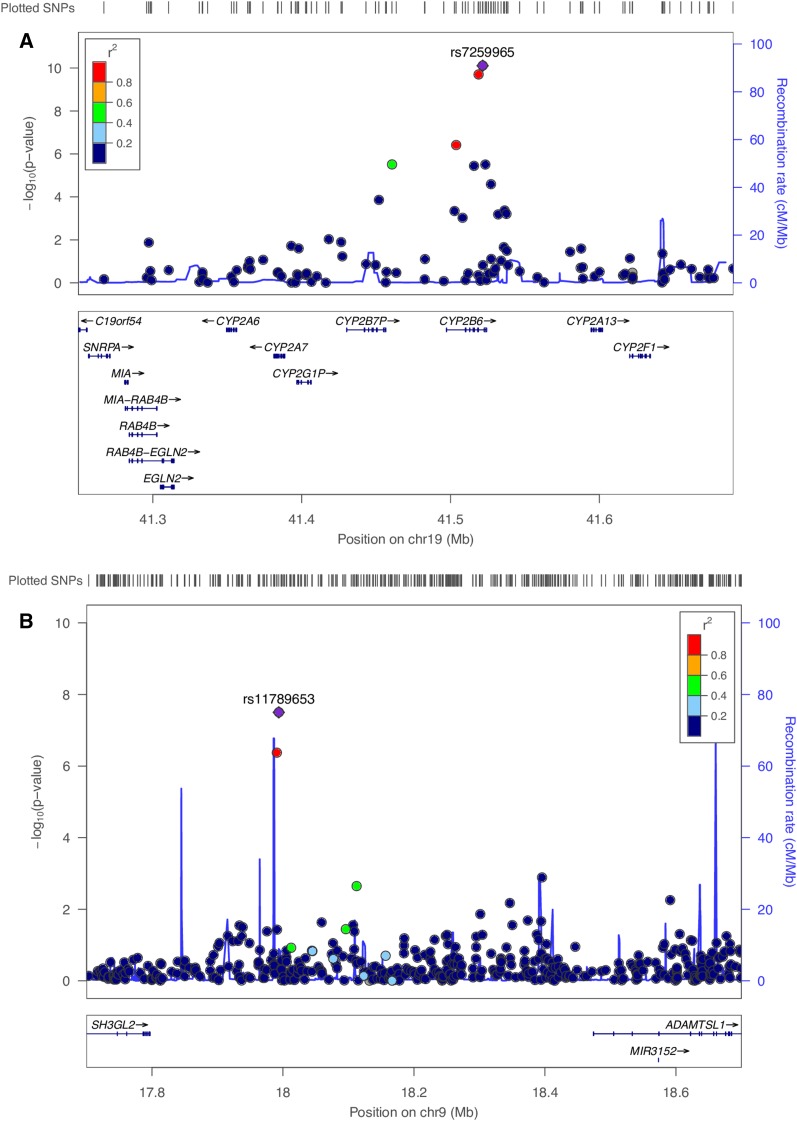
Linkage disequilibrium regional genomic plot of maternal genome-wide associated SNPs with congener serum levels. (A) rs7259965 on chromosome 19q13.2 associated with p,p′-DDE levels (β = 0.32, SE = 0.05, *P* = 7.8 × 10^−11^) maps to the *CYP2B6* gene; (B) rs11789653 on chromosome 9p22.2 associated with BDE-28 (β = 0.65, SE = 0.12, *P* = 3.2 × 10^−8^) maps between *ADAMTSL1* and *SH3GL2* genes. The *X*-axis represents the genomic position; the *Y*-axis shows the negative logarithm of the observed association *P*-value for each tested SNP. Plotted with Locuzoom tool (Pruim *et al.* 2011).

Maternal genome-wide association via linear regression identified a common SNP on chromosome 19q13.2, rs7259965, that reached genome-wide significance for p,p′-DDE (β = 0.32, SE = 0.05, *P* = 7.8 × 10^−11^) and BDE-99 (β = 0.32, SE = 0.06, *P* = 2.1 × 10^−8^) (Tables S6 in File S1) and nominally significant association (*P* < 0.05) with other PBDE congeners (Table 4), except BDE-153. This SNP maps to the *CYP2B6* gene (Figure 1A) which encodes a member of the cytochrome P450 superfamily of lipase oxidase.

**Table 4:** Genome-wide and nominally significant association of maternal chr9:rs11789653 and chr19:rs7259965 across congeners

	chr19:rs7259965 – MAF = 0.29				chr9:rs11789653 – MAF = 0.025			
Compound	*N*	β	SE	*P*-Value	*N*	β	SE	*P*-Value
Persistent pesticides								
p,p′-DDE	**759**	**0.32**	**0.05**	**7.8 × 10^−11^**	762	0.32	0.15	2.9 × 10^−2^
Polybrominated diphenyl ethers								
BDE-28	695	0.14	0.04	7.0 × 10^−4^	**698**	**0.65**	**0.12**	**3.2 × 10^−8^**
BDE-47	759	0.23	0.06	1.6 × 10^−4^	762	0.68	0.17	8.8 × 10^−5^
BDE-99	**738**	**0.32**	**0.06**	**2.1 × 10^−8^**	741	0.5	0.17	4.7 × 10^−3^
BDE-100	*747*	*0.28*	*0.06*	*3.9* × *10^−6^*	750	0.66	0.18	2.9 × 10^−4^
BDE-153	746	0.11	0.06	NS	749	0.52	0.18	3.4 × 10^−3^
Sum PBDE	707	0.24	0.05	8.2 × 10^−6^	715	0.59	0.16	3.1 × 10^−4^
Polychlorinated biphenyls								
PCB118	711	0.06	0.03	NS	715	0.2	0.1	3.7 × 10^−2^
PCB153	752	0.04	0.03	NS	755	0.21	0.1	3.0 × 10^−2^

In each category, the results are shown in alphanumeric order. The genome-wide significant associations are shown in **bold** and the suggestively significant associations are in *italics*. Genome-wide association threshold is *P* = 5 × 10^−8^ and suggestive association threshold is *P* = 5 × 10^−6^. MAF, minor allele frequency; SE, standard error; NS, not significant.

A second maternal SNP was associated with several PBDE congeners: low-frequency variant rs11789653 (MAF = 0.025) reached genome-wide significance for low-detected BDE-28 (β = 0.65, SE = 0.12, *P* = 3.2 × 10^−8^) (Table S6 in File S1) and showed nominal significance with other PBDEs, p,p′-DDE, PCB118, and PCB153 (Table 4). The marker rs11789653 resides on chromosome 9p22.2 between *ADAMTSL1* and *SH3GL2* (Figure 1B): ADAMTSL1 or punctin is a secreted molecule resembling members of the ADAMTS (a disintegrin-like and metalloprotease domain with thrombospondin type-1 repeats) family of proteases and *SH3GL2* [Src homology 3 (SH3) domain GRB2-like 2] encodes the endophilin-1 protein. BDE-100, BDE-153, and SumPBDE, which were negatively and significantly associated with offspring ASD outcome, did not show genome-wide significant association but demonstrated suggestive or nominal association with two SNPs (rs2855812: BDE-100, β = −0.35, SE = 0.07, *P* = 2.9 × 10^−7^; Sum PBDE, β = −0.29, SE = 0.06, *P* = 1.8 × 10^−6^, rs17506613: BDE-153, β = −0.27, SE = 0.06, *P* = 1.1 × 10^−6^) that map to *MICB* and *TACC1* genes, respectively (Table S6 in File S1).

The sum of PCB congeners showed an association near genome-wide significance with the low-frequency SNP rs35560597 that maps near *FST* and *NDUFS4* on chromosome 5q11.2 (β = 0.79, SE = 0.15, *P* = 7.9 × 10^−8^) (Figure S4a and Table S6 in File S1), with the most significant individual congener effect for PCB28 (*P* = 3.5 × 10^−6^). Other loci showed suggestive association (*P* < 5.0 × 10^−7^) mainly with PCB congeners (Tables S6 and S7 in File S1): rs75107653 (PCB196/203, β = 0.57, SE = 0.11, *P* = 8.6 × 10^−8^) maps near *SETMAR* and *SUMF1* on chromosome 3p26.1; rs4945829 (PCB196/203, β = −0.16, SE = 0.03, *P* = 1.1 × 10^−7^) maps adjacent to the *CCDC162P* gene and near the *CEP57L1* and *CD164* genes on chromosome 6q21, and rs10500669 (PCB187, β = 0.20, SE = 0.04, *P* = 2.5 × 10^−7^) is located on chromosome 11p15.4 near the *PRKCDBP* gene (Figure S4, b–d and Table S6 in File S1). BB-153 is suggestively associated with a locus on chromosome 18p11.31 (rs56385737, β = 0.44, SE = 0.08, *P* = 3.1 × 10^−7^), which maps to the *DLGAP1* gene (Figure S4e and Table S6 in File S1).

### Fetal SNP-based heritability of midgestational organohalogen concentrations

We calculated the contribution of fetal genetic factors to the variability of midgestational maternal levels of organohalogens by utilizing neonatal DNA samples. Fetal heritability of PBDEs (66–99%) exceeded maternal estimates (39–93%) but similarly high SEs occurred due to the small sample size (Table 2). There were two significant fetal heritability estimates for PCBs, one for PCB153 (*h*^2^*_g_* = 0.58, SE = 0.34, *P* = 0.04) and one for PCB138/158 (*h*^2^*_g_* = 0.57, SE = 0.34, *P* = 0.04) (Table 2). BDE-153 showed significant fetal genetic correlations with BDE-28, -47, and -99, which were higher than estimated maternal genetic correlations (Table 3). However, some pairwise genetic correlations could not be calculated due to the small sample size (Table 3).

### Fetal genome-wide association analysis of midgestational organohalogen concentrations

**Figure 2:**
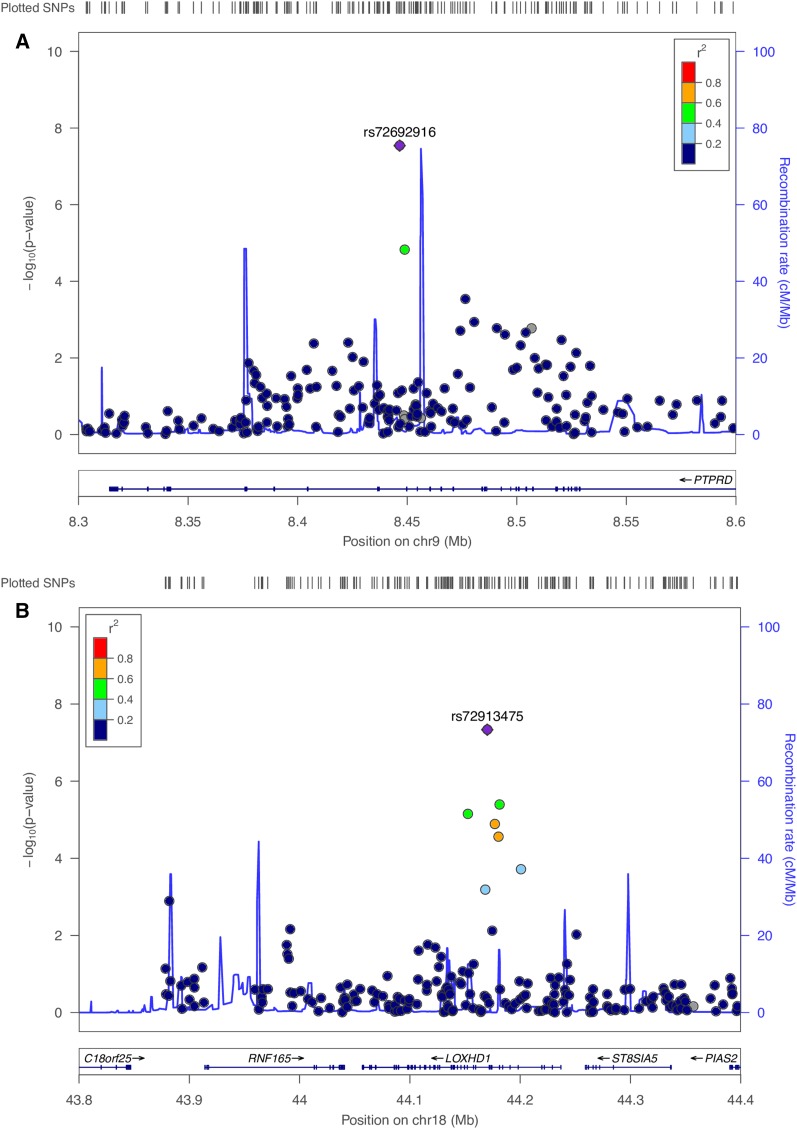
Linkage disequilibrium regional genomic plot of fetal genome-wide associated SNPs with congener serum levels. (A) rs72692916 on chromosome 9p24.1 associated with PCB187 (β = −0.42, SE = 0.08, *P* = 2.8 × 10^−8^); maps to the *PTPRD* gene; (B) rs72913475 on chromosome 18q21.1 associated with BDE-100 (β = 0.55, SE = 0.1, *P* = 4.6 × 10^−8^) maps to the *LOXHD1* gene. The *X*-axis represents the genomic position; the *Y*-axis shows the negative logarithm of the observed association *P*-value for each tested SNP. Plotted with Locuzoom tool (Pruim *et al.* 2011).

Fetal genome-wide association of midgestational maternal levels of organohalogens identified one SNP, rs72692916, significantly associated with PCB187 (MAF = 0.05; β = −0.42, SE = 0.08, *P* = 2.8 × 10^−8^) and suggestively associated across PCBs except PCB28 (Figure 2A and Table S8 in File S1). This SNP is located on chromosome 9p24.1 and maps to the *PTPRD* gene. A second SNP, rs72913475, on chromosome 18q21.1 that maps to the *LOXHD1* gene was significantly associated with BDE-100 (MAF = 0.08; β = 0.55, SE = 0.1, *P* = 4.6 × 10^−8^) and showed effects with other PBDEs (Figure 2B and Table S9 in File S1). Maternal PBDEs and PCBs were also associated with several additional loci at the suggestive level in offspring DNA (Table S9 in File S1).

### Maternal and fetal contribution to the associated loci

Because we had mother–infant pairs, we were able to test whether the genetic loci significantly associated with organohalogens were controlled by maternal or fetal genetics, *i.e.*, whether we observed fetal association only because of the inheritance of maternal alleles. We selected maternal top SNPs for each organohalogen meeting *P* < 5.0 × 10^−8^ (Table S6 in File S1) that also showed nominal effects in fetal genetic associations of the same congener: rs7259965 in *CYP2B6* (p′-p′-DDE, *maternal P* = 7.8 × 10^−11^; *neonatal P* = 1.9 × 10^−4^; BDE-99, *maternal P* = 2.1 × 10^−8^; *neonatal P* = 1.3 × 10^−5^), rs11789653 near *ADAMTSL1-SH3GL2* (BDE-28, *maternal P* = 3.2 × 10^−8^; *neonatal P* = 4.7 × 10^−6^), rs75107653 near *SETMAR-SUMF1* (PCB196/203, *maternal P* = 8.6 × 10^−8^; *neonatal P* = 2.0 × 10^−4^), and rs10500669 near *PRKCDBP* (PCB187, *maternal P* = 2.5 × 10^−7^; *neonatal P* = 1.6 × 10^−3^) were used. Additionally, we selected one fetal top SNP rs72913475 in *LOXDH1* (Table S5 in File S1) that showed nominal effects in maternal genetic associations of BDE-100 (*maternal P* = 4.7 × 10^−3^; *neonatal P* = 4.6 × 10^−8^). We included the genotypes of each pair of maternal–fetal SNPs in linear models to assess whether maternal and/or fetal genetic factors showed an independent contribution to the association of each respective congener above. The maternal–fetal combined models that include genome-wide maternal-associated SNP and nominal fetal SNP showed attenuated or nonsignificant fetal association and vice versa; the combined model that includes the genome-wide fetal-associated SNP and the same nominal maternal SNP showed only significant fetal association (Table 5). We did not find evidence that maternal genetic factors contributed independently to loci detected via fetal association or vice versa, suggesting that fetal and maternal genetics have separate effects (Table 5).

**Table 5:** Combined effects of maternal–fetal SNPs in linear regression models

					Combined: Maternal SNP*^a^*			Combined: Fetal SNP*^a^*		
Compound	SNP	CHR	BP	Locus	β	SE	*P*-Value	β	SE	*P*-Value
p,p′-DDE	rs7259965	19	41,521,532	*CYP2B6*	0.3	0.05	4.4 × 10^−9^	0.05	0.05	NS
BDE-99	rs7259965	19	41,521,532	*CYP2B6*	0.33	0.06	8.8 × 10^−8^	0.11	0.06	4.1 × 10^−2^
BDE-28	rs11789653	9	17,993,589	*ADAMTSL1-SH3GL2*	0.63	0.12	4.2 × 10^−7^	0.19	0.12	NS
PCB196/203	rs75107653	3	4,181,811	*SETMAR-SUMF1*	0.59	0.11	6.8 × 10^−8^	0.04	0.11	NS
PCB187	rs10500669	11	6,328,472	*PRKCDBP*	0.19	0.04	1.5 × 10^−6^	0	0.04	NS
BDE-100	rs72913475	18	44,170,156	*LOXHD1*	0.05	0.1	NS	0.55	0.1	7.5 × 10^−8^

CHR, chromosome; BP, position; SE, standard error; NS, not significant.

a Linear regression models adjusted for maternal birth place, maternal age, maternal education attainment, offspring gender, month of birth, parity, 10 ancestry coordinates, offspring ASD outcome status, maternal SNP genotypes, and fetal SNP genotypes.

### Effect of maternal and fetal PBDE genetic factors on ASD outcome

We expected that the genetic factors that contribute to BDE-100, -153, and Sum PBDE might drive the association with ASD outcome (Table 1). After the inclusion of individual estimates of the maternal and fetal total additive genetic effects (summarized in a BLUP) in the linear regression models, we found strongly attenuated or nonsignificant association between ASD outcome and PBDEs, supporting this hypothesis (Table 6). In order to investigate whether individual loci could account for this attenuation, we considered the most associated maternal and fetal SNPs for BDE-100 and BDE-153. Since no maternal genome-wide significant associated loci emerged for BDE-100 and BDE-153 and only one genome-wide (GW) fetal locus is associated with BDE-100 (Tables S6 and S9 in File S1), we selected two maternal SNPs (rs2855812: BDE-100, β = −0.35, SE = 0.07, *P* = 2.9 × 10^−7^, rs17506613: BDE-153, β = −0.27, SE = 0.06, *P* = 1.1 × 10^−6^) and one fetal SNP (rs72913475: BDE-100, β = 0.55, SE = 0.10, *P* = 4.6 × 10^−8^) most strongly associated with congeners related to ASD outcome to determine whether they might explain the negative relationship between PBDEs and ASD. We excluded Sum PBDE from our analysis because we are not able to easily interpret any finding that links the sum of congener levels with ASD outcome. These SNPs were not themselves associated in logistic models with ASD outcome, and they did not account for the association between maternal PBDE congeners and ASD outcome in offspring (*P* > 0.1, each). No significant ASD by SNP interactions were identified (data not shown). However, given that the three associated SNPs accounted for a small proportion of each PBDE level variance (rs2855812 σ = 3.0%; rs17506613 σ = 2.5%, rs72913475 σ = 3.7%), we calculated that our case–control sample size has a power at 80% (α = 0.05) of detecting an OR approximately below 0.3 (strong protective effect) for ASD per each PBDE unit. Our case–control analysis is thus underpowered to draw strong conclusions about individual SNP–congener relationships because the estimated ORs for ASD per unit were 0.82 and 0.84 for BDE-153 and -100, respectively (Table 1).

**Table 6:** ASD outcome association with PBDE congeners in mother and newborn datasets after maternal and fetal BLUP adjustment

	Mothers				Newborns			
Congener	*N*	β	SE	*P*-Value	*N*	β	SE	*P*-Value
BDE-100	750	−0.17	0.07	2.1 × 10^−2^	697	−0.17	0.08	2.8 × 10^−2^
BDE-100 w BLUP	750	−0.004	0.002	0.051	697	−0.0006	0.002	NS
BDE-153	749	−0.19	0.07	6.9 × 10^−3^	695	−0.19	0.07	9.9 × 10^−3^
BDE-153 w BLUP	749	−0.003	0.003	NS	695	−0.0003	0.001	NS
Sum PBDE	710	−0.16	0.07	1.8 × 10^−2^	659	−0.16	0.07	2.3 × 10^−2^
Sum PBDE w BLUP	710	−0.009	0.004	4.6 × 10^−2^	659	−0.002	0.002	NS

BLUP, best linear unbiased prediction; SE, standard error; NS, not significant. *P* < 0.1.

## Discussion

We report here the first large-scale genetic study of the maternal peripheral blood levels of several environmentally persistent and potential neurotoxic organohalogen compounds measured in midpregnancy, among a diverse-ancestry population of women. We first assessed whether maternal circulating levels of organohalogens were regulated by maternal genetic factors. For the first time, we found evidence that a large proportion of maternal circulating levels of BB-153, BDE-47, -100, -153, and their sum was significantly controlled by common genetic factors [*h*^2^*_g_* (73–99%)]. Additionally, some pairs of PBDE congeners showed genetic correlation significantly higher than linear correlation, indicating that even when the exposure sources and/or the contribution of diet and lifestyle to the pollutant accumulation in the body differ between organohalogens, shared genetic factors can affect the observed circulating levels, *e.g.*, metabolism or lipid levels.

In GWAS analysis to identify the maternal genetic factors that regulate circulating levels of midgestational organohalogens, maternal genome-wide significant associations between p,p′-DDE and PBDE levels and a SNP which maps within the *CYP2B6* locus were found. Our results suggest that the encoded P450 2B6 enzyme may influence the levels of circulating organohalogens, but the function of the associated SNP rs7259965 (or its LD proxies) on enzyme expression or function is unknown. The encoded protein is a member of the cytochrome P450 superfamily of enzymes that catalyze reactions involved in xenobiotic metabolism and drug pharmacokinetics, in synthesis of cholesterol, steroids, and other lipids. Previous evidence of association between CYP genes and ASD or autistic traits has been reported in studies motivated by sex-hormone biosynthesis but not including environmental biomarkers (Chakrabarti *et al.* 2009). A study (Lind *et al.* 2016) published when this paper was under review highlighted a significant association between rs7260538 in *CYP2B6* and p,p′-DDE levels measured in a Swedish cohort of 1016 elderly individuals and, after conditional analysis using the lead SNP, a second SNP, rs7255374, showed an independent genome-wide significant association (*P* = 2.2 × 10^−8^). We did not genotype the published SNPs but based on a 1000 Genome reference panel, our associated SNP (rs7259965) is in low LD with rs7260538 (*r*^2^ < 0.4) and in high LD with rs7255374, the second independent associated SNP (*r*^2^ between 0.6 and 0.8). Recently, SNPs in the *CYP2B6* gene were also associated with the circulating levels of BDE-47 in 1500 elderly individuals (Penell *et al.* 2014). Enzymes in this class are known to act as mediators of the oxidative transformation of PCB (Gährs *et al.* 2013; Lehmler *et al.* 2010) and PBDE congeners into hydroxylated compounds in the liver. In particular, P450 2B6, encoded by *CYP2B6*, emerged as the most active enzyme.

Additionally, Ng *et al.* (2015) showed a genome-wide association between rs8109848 (β = 0.34, SE = 0.05, *P* = 3.7 × 10^−13^) in *CYP2B6* and PCB levels in the same Swedish cohort. We did not genotype the published SNP and we only found nominally significant association across PCBs with rs7246465 in the opposite direction (*r*^2^ > 0.4 linkage disequilibrium with rs8109848). The PBDE-associated *CYP2B6* SNP rs7259965 was only nominally significant for the sum of PCB congeners (Sum PCB; β = 0.08, SE = 0.04, *P* = 0.02). Our sample could be underpowered to find *CYP2B6* association with PCB levels or the differences in enrolled subjects between the Swedish study and ours (both genders >70 yr old *vs.* females-only <45 yr old) might explain lack of replication, particularly given that PCB levels have shown differences across gender (Salihovic *et al.* 2012) and age (Needham *et al.* 2006).

Our results also highlighted maternal near genome-wide significant associations between PCB levels and SNPs that map within or near loci involved in hormone or lipid metabolism: *SUMF1* encodes the sulfatase-modifying factor 1, the key activator of sulfatases that regulate sulfate homeostasis, a fundamental biologic process for fetal development and hormone metabolism (Dawson *et al.* 2013; Settembre *et al.* 2007). The protein kinase C δ binding protein PRKCDBP is a candidate tumor suppressor gene and a transcription target of TNF-α, a critical proinflammatory cytokine, which plays a crucial role in colonic inflammation and tumorigenesis and is involved in the regulation of circadian clock components and metabolic syndrome (Kovanen *et al.* 2014). FST or follistatin is secreted by the liver based on the glucagon-to-insulin ratio (Hansen *et al.* 2016) and inhibits follicle-stimulating hormone release. An SNP near *NDUFS4* was associated with the sum of PCBs and encodes a member of the complex I of the mitochondrial respiratory chain NADH dehydrogenase included in the metformin pathway in liver cells. Metformin is known (Ota *et al.* 2009; Owen *et al.* 2000; El-Mir *et al.* 2000) to inhibit the respiratory complex I and consequently, increase the cellular AMP:ATP ratios that may activate AMPK, a major cellular regulator of lipid and glucose metabolism (Owen *et al.* 2000; Hardie 2006)

Based on previous evidence that fetal genotypes may affect processes that alter pre- and postnatal maternal physiology (Liu *et al.* 2015a; Petry *et al.* 2007, 2014), we hypothesized that the circulating levels of maternal organohalogens could also be influenced by fetal genetic factors that might play an active role in controlling the toxicant disposition between mother and fetus. Surprisingly, we found that the heritability and genetic correlation results for midgestational levels of BB-153, BDE-47, -99, -100, -153, and Sum PBDE showed evidence for a higher impact of the total fetal genome-wide SNP-based genetic effects on maternal compound levels than the total maternal genetic effects, using equivalent datasets. These results suggest that the maternal circulating levels of some compounds were more highly influenced by fetal genetic factors than maternal genetics. When assessing the association between maternal circulating organohalogens and fetal SNPs, we found two loci that reached genome-wide significance: the first SNP associated with PCB187 maps to the *PTPRD* gene that encodes the tyrosine phosphatase receptor δ that regulates learning processes and synaptic plasticity and has recently been identified as a novel locus for ASD in Asians (Liu *et al.* 2015b). PTPRD may localize in axons and in dendrites to regulate their elaboration in the central nervous system (Shishikura *et al.* 2016) and it showed moderate expression in fetal trophoblasts of the placenta (see www.proteinatlas.org/ENSG00000153707-PTPRD/tissue/primary+data). The second BDE-100-associated locus maps to the *LOXHD1* gene (Lipoxygenase Homology Domains 1) that encodes a protein that consists of PLAT (polycystin/lipoxygenase/alpha-toxin) domains (Bateman and Sandford 1999), thought to be involved in targeting proteins to the plasma membrane (Aleem *et al.* 2009). We did not find evidence of independent maternal contribution in fetal-driven associations or vice versa. Our findings showed that the strong genetic basis for midgestational circulating organohalogen levels includes contributions from a set of nonoverlapping maternal-driven and fetal-driven genetic factors, which we could then utilize to follow up association between circulating chemicals and ASD.

We confirmed that the serum levels of BDE-100, -153, and the total sum of PBDEs were significantly lower in mothers of ASD-affected children compared to mothers of control children, as reported in the full EMA study (Tsang *et al.* 2013). In the original EMA study sample, we highlighted positive associations between the highest quartile (relative to the lowest quartile) of PCB 138/158 and PCB 153 and risk of ASD (Lyall *et al.* 2015). In the genotyped subset of the EMA sample studied here, examining continuously modeled PCB levels, we did not find association between maternal PCB congeners and ASD risk (*P* > 0.1). The difference is due to the models used (continuous levels *vs.* quartiles), as we observed consistent results using the quartile approach in the genotyped EMA dataset (Table S1 in File S1). Additionally, we confirmed that the inclusion in our regression models of more than two genetic ancestry coordinates and the exclusion of the extreme values of distributions did not affect the association between PCBs and ASD.

When we examined the associated PBDE congeners and accounted for genome-wide additive genetic effects predicting their levels, the negative associations between PBDEs and ASD outcome were no longer observed. Although no specific single PBDE-associated SNP explained this result, overall it suggests that a large set of low-impact genetic determinants of PBDE levels are also at least partly responsible for the previously observed association with ASD outcome. Based on these findings, we hypothesized several possible scenarios to explain the negative association between circulating PBDEs and ASD outcome in offspring. First, our evidence showed that at least some associated maternal genetic factors involved in controlling PBDE levels map within or between genes implicated in metabolism of xenobiotic compounds. Thus, a negative association could be driven by toxic metabolites that might be produced by breakdown of organohalogens and negatively correlated with circulating levels of the presumed toxicants. Alternatively, the observation of a negative association between maternal levels of a presumed toxicant and ASD outcome in offspring could be due to pleiotropy of pathways involved in xenobiotic metabolism, with the true risk factors being other substances metabolized by the same enzymes, but, for example, with different affinity. Second, we observed that genes in fetal genetic loci contributing to levels of organohalogens in the maternal bloodstream showed moderate expression in the fetal compartment of placenta (*i.e.*, *PTPRD*). Thus, transplacental transfer of organohalogens during pregnancy may be driven by the fetal genome expressed in placenta (Bianco-Miotto *et al.* 2016), partially driving maternal physiologic and metabolic phenotypic changes during pregnancy as previously described (*e.g.*, blood pressure) (Liu *et al.* 2015a; Petry *et al.* 2007, 2014). Previous findings showed significant positive correlations between maternal blood concentrations of organohalogens and cord blood after the first trimester (Zhao *et al.* 2013; Vizcaino *et al.* 2014) of pregnancy and between maternal levels and umbilical cord serum at 6 months of pregnancy (Choi *et al.* 2014) and at delivery (Hernik *et al.* 2013) providing evidence of the placental transfer of organohalogens between mother and fetus. High maternal-fetal transfer mean ratios were shown for BDE-47 and BDE-99 in a placenta perfused model but a small overall amount was found in fetus (Frederiksen *et al.* 2010). However, one study observed that the fetus is exposed to PBDEs from at least the second trimester of pregnancy to birth (Foster *et al.* 2011), indicating that the transfer to the fetus could be specific to chemical properties such as molecular weight, lipid solubility, and protein binding (Myllynen *et al.* 2009). We also suggest that transfer may be influenced by the fetal genetic factors of placenta such as variation in transport efficiency and lipid stores. Our results suggest that the circulating levels of organohalogens are associated with genetic factors that might drive the maternal–fetal transfer but we are not able to directly support a hypothesis for the negative association observed between blood levels of PBDEs and ASD outcome. We also speculate that the maternal serum levels of PBDEs account for only a low proportion of the total PBDE accumulated in other biological matrices, such as adipose-rich tissues, and these PBDEs are postnatally transferred to newborns via maternal breast milk (Antignac *et al.* 2009). However, we did not measure organohalogen levels at birth in maternal or neonatal samples or in other biological matrices.

### Strengths and limitations

Major strengths of this study include a mother–infant paired sample of varied ancestry, the measurement of chemical environmental pollutants in blood of pregnant women during a gestational time (15–20 wk) relevant to neurodevelopment, and diagnostic confirmation of ASD status. Additionally, the availability of maternal and neonatal genotypes, several confounding factors that predict the levels of exposure, and the ancestry genetic coordinates allowed us to analyze the maternal and neonatal genetic factors that may regulate the levels of circulating environmental chemicals in the maternal bloodstream. Limitations include the reduced power of our sample size to detect moderate heritability and to assess the causality between PBDEs and ASD, the generalization of our findings from the target populations to other populations, the replacement of measurements below the LOD, and the low limit of detection of BDE-28 as well as lack of measurement of other potential confounders such as BMI or diet. Although we have used significance thresholds appropriate for genome-wide testing per biomarker, we have performed testing across a large number of biomarkers and for both maternal and fetal genomes, so any individual results require independent replication.

### Conclusions

Our evidence suggests that maternal and fetal genetic make-up are important determinants of midgestational maternal circulating levels of some environmental organohalogens. Previous results showed that organohalogen levels were associated with metabolic dysfunction, including obesity (Vuong *et al.* 2016; Frugé *et al.* 2016), and PCB levels were associated with gestational and type 2 diabetes (Jaacks *et al.* 2016; Esser *et al.* 2016) and Parkinson’s disease (Hatcher-martin *et al.* 2012; Petersen *et al.* 2008). Our results strongly suggest that the genes involved in xenobiotic and lipid metabolism such as *CYP2B6* may be involved in controlling PBDE levels and confirm the association with persistent organochlorine pesticides (OCP) levels. Additional candidate loci, such as *PRKCDBP*, *SUMF1*, *NDUFS4*, and *FST*, that encode proteins important for hormone, liver, and lipid function may be involved in PCB level regulation. This study also provides evidence that fetal genetic loci near potential metabolic genes such as *LOXHD1* and genes previously implicated in neurodevelopment, such as *PTPRD*, contribute to maternal biomarker levels independent of maternal genotype. The functional relevance of these fetal loci or the second significant maternal locus, near *SH3GL2*, for circulating chemical levels is unknown. Additional work will be required to uncover novel roles for these genes or other functional elements in these regions. Future studies investigating associations between environmental exposures and metabolic disease or developmental outcomes should therefore consider maternal and fetal genetic determinants of what are thought to be environmental biomarkers.

## Supplementary Material

Supplemental material is available online at www.g3journal.org/lookup/suppl/doi:10.1534/g3.117.039784/-/DC1.

## References

[bib1] Abrahams B. S. Geschwind D. H., 2008 Advances in autism genetics: on the threshold of a new neurobiology. Nat. Rev. Genet. 9: 341–355. 18414403 10.1038/nrg2346 PMC2756414

[bib2] Affymetrix, 2011 *Analysis Guide Axiom Genotyping Solution Data Analysis Guide. Analysis: 55. Affymetrix Power Tools* Affymetrix Inc., Santa Clara. Available at: www.affymetrix.com.

[bib3] Aleem A. M. Wells L. Jankun J. Walther M. Kuhn H., 2009 Human platelet 12-lipoxygenase: naturally occurring Q261/R261 variants and N544L mutant show altered activity but unaffected substrate binding and membrane association behavior. Int. J. Mol. Med. 24: 759–764. 19885615 10.3892/ijmm_00000289

[bib4] Antignac J. P. Cariou R. Zalko D. Berrebi A. Cravedi J. P., 2009 Exposure assessment of French women and their newborn to brominated flame retardants: determination of tri- to deca- polybromodiphenylethers (PBDE) in maternal adipose tissue, serum, breast milk and cord serum. Environ. Pollut. 157: 164–173. 18804904 10.1016/j.envpol.2008.07.008

[bib5] Autism and Developmental Disabilities Monitoring Network Surveillance Year 2000 Principal Investigators; Centers for Disease Control and Prevention, 2007 Prevalence of autism spectrum disorders – autism and developmental disabilities monitoring network, six sites, United States, 2000. MMWR Surveill. Summ. 56: 1–11. 17287714

[bib6] Axelrad D. A. Goodman S. Woodruff T. J., 2009 PCB body burdens in US women of childbearing age 2001–2002: an evaluation of alternate summary metrics of NHANES data. Environ. Res. 109: 368–378. 19251256 10.1016/j.envres.2009.01.003

[bib7] Bateman A. Sandford R., 1999 The PLAT domain: a new piece in the PKD1 puzzle. Curr. Biol. 9: R588–R590. 10469604 10.1016/s0960-9822(99)80380-7

[bib8] Bauman M. L. Kemper T. L., 2005 Neuroanatomic observations of the brain in autism: a review and future directions. Int. J. Dev. Neurosci. 23: 183–187. 15749244 10.1016/j.ijdevneu.2004.09.006

[bib9] Bianco-Miotto T. Mayne B. Buckberry S. Breen J. Rodriguez Lopez C. R. C., 2016 Recent progress towards understanding the role of DNA methylation in human placental development. Reproduction. 152: R23–R30. 27026712 10.1530/REP-16-0014 PMC5064761

[bib10] Bilbo S. D. Schwarz J. M., 2012 The immune system and developmental programming of brain and behavior. Front. Neuroendocrinol. 33: 267–286. 22982535 10.1016/j.yfrne.2012.08.006 PMC3484177

[bib11] Braun J. M. Kalkbrenner A. E. Just A. C. Yolton K. Calafat A. M., 2014 Gestational exposure to endocrine-disrupting chemicals and reciprocal social, repetitive, and stereotypic behaviors in 4- and 5-year-old children: The HOME study. Environ. Health Perspect. 122: 513–520. 24622245 10.1289/ehp.1307261 PMC4014765

[bib12] Brion M. J. A. Shakhbazov K. Visscher P. M., 2013 Calculating statistical power in Mendelian randomization studies. Int. J. Epidemiol. 42: 1497–1501. 24159078 10.1093/ije/dyt179 PMC3807619

[bib13] Chakrabarti B. Dudbridge F. Kent L. Wheelwright S. Hill-Cawthorne G., 2009 Genes related to sex steroids, neural growth, and social-emotional behavior are associated with autistic traits, empathy, and Asperger syndrome. Autism Res. 2: 157–177. 19598235 10.1002/aur.80

[bib14] Choi G. Kim S. Kim S. Kim S. Choi Y., 2014 Occurrences of major polybrominated diphenyl ethers (PBDEs) in maternal and fetal cord blood sera in Korea. Sci. Total Environ. 491–492: 219–226. 10.1016/j.scitotenv.2014.02.071 24636800

[bib15] Choksi N. Y. Kodavanti P. R. Tilson H. A Booth R. G., 1997 Effects of polychlorinated biphenyls (PCBs) on brain tyrosine hydroxylase activity and dopamine synthesis in rats. Fundam. Appl. Toxicol. 39: 76–80. 9325030 10.1006/faat.1997.2351

[bib16] Croen L. A. Grether J. K. Hoogstrate J. Selvin S., 2002 The changing prevalence of autism in California. J. Autism Dev. Disord. 32: 207–215. 12108622 10.1023/a:1015453830880

[bib17] Croen L. A. Goines P. Braunschweig D. Yolken R. Yoshida C. K., 2008 Brain-derived neurotrophic factor and autism: maternal and infant peripheral blood levels in the early markers for autism (EMA) study. Autism Res. 1: 130–137. 19119429 10.1002/aur.14 PMC2613010

[bib18] Dawson S.-J. Tsui D. W. Y. Murtaza M. Biggs H. Rueda O. M., 2013 Analysis of circulating tumor DNA to monitor metastatic breast cancer. N. Engl. J. Med. 368: 1199–1209. 23484797 10.1056/NEJMoa1213261

[bib19] Debost-Legrand A. Warembourg C. Massart C. Chevrier C. Bonvallot N., 2016 Prenatal exposure to persistent organic pollutants and organophosphate pesticides, and markers of glucose metabolism at birth. Environ. Res. 146: 207–217. 26775002 10.1016/j.envres.2016.01.005

[bib20] El-Mir M.-Y. Nogueira V. Fontaine E. Avéret N. Rigoulet M., 2000 Dimethylbiguanide inhibits cell respiration via an indirect effect targeted on the respiratory chain complex I. J. Biol. Chem. 275: 223–228. 10617608 10.1074/jbc.275.1.223

[bib21] Elliott P. Chambers J. C. Zhang W. Clarke R. Hopewell J. C., 2009 Genetic loci associated with C-reactive protein levels and risk of coronary heart disease. JAMA 302: 37–48. 19567438 10.1001/jama.2009.954 PMC2803020

[bib22] Eriksson P. Jakobsson E. Fredriksson A., 2001 Brominated flame retardants: a novel class of developmental neurotoxicants in our environment? Environ. Health Perspect. 109: 903–908. 11673118 10.1289/ehp.01109903 PMC1240439

[bib23] Esser A. Schettgen T. Gube M. Koch A. Kraus T., 2016 Association between polychlorinated biphenyls and diabetes mellitus in the German HELPcB cohort. Int. J. Hyg. Environ. Health 219: 557–565. 27397874 10.1016/j.ijheh.2016.06.001

[bib24] Foster W. G. Gregorovich S. Morrison K. M. Atkinson S. A. Kubwabo C., 2011 Human maternal and umbilical cord blood concentrations of polybrominated diphenyl ethers. Chemosphere 84: 1301–1309. 21663933 10.1016/j.chemosphere.2011.05.028

[bib25] Frederiksen M. Vorkamp K. Mathiesen L. Mose T. Knudsen L. E., 2010 Placental transfer of the polybrominated diphenyl ethers BDE-47, BDE-99 and BDE-209 in a human placenta perfusion system: an experimental study. Environ. Health 9: 32. 20598165 10.1186/1476-069X-9-32 PMC2908602

[bib26] Frugé A. D. Cases M. G. Schildkraut J. M. Demark-Wahnefried W., 2016 Associations between obesity, body fat distribution, weight loss and weight cycling on serum pesticide concentrations. J. Food Nutr. Disord. 5(3): 10.4172/2324-9323.1000198. 10.4172/2324-9323.1000198 PMC4966654 27478857

[bib27] Gährs M. Roos R. Andersson P. L. Schrenk D., 2013 Role of the nuclear xenobiotic receptors CAR and PXR in induction of cytochromes P450 by non-dioxinlike polychlorinated biphenyls in cultured rat hepatocytes. Toxicol. Appl. Pharmacol. 272: 77–85. 23770461 10.1016/j.taap.2013.05.034

[bib28] Gaugler T. Klei L. Sanders S. J. Bodea C. A. Goldberg A. P., 2014 Most genetic risk for autism resides with common variation. Nat. Genet. 46: 881–885. 25038753 10.1038/ng.3039 PMC4137411

[bib29] Goines P. Haapanen L. Boyce R. Duncanson P. Braunschweig D., 2011 Autoantibodies to cerebellum in children with autism associate with behavior. Brain Behav. Immun. 25: 514–523. 21134442 10.1016/j.bbi.2010.11.017 PMC3039058

[bib30] Grandjean P. Landrigan P. J., 2014 Neurobehavioural effects of developmental toxicity. Lancet Neurol. 13: 330–338. 24556010 10.1016/S1474-4422(13)70278-3 PMC4418502

[bib31] Hansen J. S. Rutti S. Arous C. Clemmesen J. O. Secher N. H., 2016 Circulating follistatin is liver-derived and regulated by the glucagon-to-insulin ratio. J. Clin. Endocrinol. Metab. 101: 550–560. 26652766 10.1210/jc.2015-3668

[bib32] Hardie D. G., 2006 Neither LKB1 nor AMPK are the direct targets of metformin. Gastroenterology 131: 973. 16952573 10.1053/j.gastro.2006.07.032

[bib33] Hatcher-martin J. M. Gearing M. Steenland K. Levey A. I. Miller G. W., 2012 NeuroToxicology association between polychlorinated biphenyls and Parkinson’s disease neuropathology. Neurotoxicology 33: 1298–1304. 22906799 10.1016/j.neuro.2012.08.002 PMC3470755

[bib34] Hernik A. Góralczyk K. Struciński P. Czaja K. Korcz W., 2013 Polybrominated diphenyl ethers and polychlorinated biphenyls in cord blood from women in Poland. Chemosphere 93: 526–531. 23856467 10.1016/j.chemosphere.2013.06.045

[bib35] Jaacks L. M. Barr D. B. Sundaram R. Maisog J. M. Zhang C., 2016 Pre-pregnancy maternal exposure to polybrominated and polychlorinated biphenyls and gestational diabetes: a prospective cohort study. Environ. Health 15: 11. 26792546 10.1186/s12940-016-0092-5 PMC4721055

[bib36] Korrick S. A. Sagiv S. K., 2008 Polychlorinated biphenyls, organochlorine pesticides and neurodevelopment. Curr. Opin. Pediatr. 20: 198–204. 18332718 10.1097/MOP.0b013e3282f6a4e9 PMC3878996

[bib37] Kovanen L. Donner K. Kaunisto M. Partonen T., 2015 CRY1, CRY2 and PRKCDBP genetic variants in metabolic syndrome. Hypertens. Res. 38: 186–192. 25391456 10.1038/hr.2014.157

[bib38] Lee S. H. Yang J. Goddard M. E. Visscher P. M. Wray N. R., 2012 Estimation of pleiotropy between complex diseases using single-nucleotide polymorphism-derived genomic relationships and restricted maximum likelihood. Bioinformatics 28: 2540–2542. 22843982 10.1093/bioinformatics/bts474 PMC3463125

[bib39] Legler J. Brouwer A., 2003 Are brominated flame retardants endocrine disruptors? Environ. Int. 29: 879–885. 12850103 10.1016/S0160-4120(03)00104-1

[bib40] Lehmler H. J. Harrad S. J. Hühnerfuss H. Kania-Korwel I. Lee C. M., 2010 Chiral polychlorinated biphenyl transport, metabolism, and distribution: a review. Environ. Sci. Technol. 44: 2757–2766. 20384371 10.1021/es902208u PMC2855137

[bib41] Leonetti C. Butt C. M. Hoffman K. Miranda M. L. Stapleton H. M., 2016 Concentrations of polybrominated diphenyl ethers (PBDEs) and 2,4,6-tribromophenol in human placental tissues. Environ. Int. 88: 23–29. 26700418 10.1016/j.envint.2015.12.002 PMC4755871

[bib42] Lilienthal H. Hack A. Roth-Härer A. Grande S. W. Talsness C. E., 2006 Effects of developmental exposure to 2,2, 4,4, 5-pentabromodiphenyl ether (PBDE-99) on sex steroids, sexual development, and sexually dimorphic behavior in rats. Environ. Health Perspect. 114: 194–201. 16451854 10.1289/ehp.8391 PMC1367831

[bib43] Lind L. Ng E. Ingelsson E. Lindgren C. Salihovic S., 2016 Genetic and methylation variation in the CYP2B6 gene is related to circulating p,p′-dde levels in a population-based sample. Environ. Int. 98: 212–218. 27839851 10.1016/j.envint.2016.11.010 PMC5152752

[bib44] Liu N. Archer E. Srinivasasainagendra V. Allison D. B., 2015 a A statistical framework for testing the causal effects of fetal drive. Front Genet. 5: 464. 10.3389/fgene.2014.00464 PMC4292723 25628644

[bib45] Liu X. Shimada T. Otowa T. Wu Y. Y. Kawamura Y., 2015 b Genome-wide association study of autism spectrum disorder in the East Asian populations. Autism Res. 9: 340–349. 26314684 10.1002/aur.1536

[bib47] Lyall K. Croen L. A. Zerbo O. Yoshida C. K. Kharrazi M., 2016 Polychlorinated biphenyl and organochlorine pesticide concentrations in maternal mid-pregnancy serum samples: association with autism spectrum disorder and intellectual disability. Environ. Health Perspect. 1–35 DOI:10.1289/EHP277. 10.1289/EHP277 PMC5332182 27548254

[bib48] McDonald T. A., 2002 A perspective on the potential health risks of PBDEs. Chemosphere 46: 745–755. 11999798 10.1016/s0045-6535(01)00239-9

[bib49] Mead J. Ashwood P., 2015 Evidence supporting an altered immune response in ASD. Immunol. Lett. 163: 49–55. 25448709 10.1016/j.imlet.2014.11.006

[bib50] Mitchell M. M. Woods R. Chi L. H. Schmidt R. J. Pessah I. N., 2012 Levels of select PCB and PBDE congeners in human postmortem brain reveal possible environmental involvement in 15q11-q13 duplication autism spectrum disorder. Environ. Mol. Mutagen. 53: 589–598. 22930557 10.1002/em.21722 PMC3739306

[bib51] Myllynen P. Immonen E. Kummu M. Vähäkangas K., 2009 Developmental expression of drug metabolizing enzymes and transporter proteins in human placenta and fetal tissues. Expert Opin. Drug Metab. Toxicol. 5: 1483–1499. 19785513 10.1517/17425250903304049

[bib52] Needham L. Patterson D. G. Jr Calafat A. M. Sjödin A. Turner W., 2006 Distribution of halogenated environmental chemicals among people of different ages, races, and sexes in the United States. Organohalogen Compd. 68: 484–487.

[bib53] Ng E. Salihovic S. Lind P. M. Mahajan A. Syvänen A., 2015 Genome-wide association study of plasma levels of polychlorinated biphenyls disclose an association with the CYP2B6 gene in a population-based sample. Environ. Res. 140: 95–101. 25839716 10.1016/j.envres.2015.03.022 PMC4509719

[bib54] Nowack N. Wittsiepe J. Kasper-Sonnenberg M. Wilhelm M. Schölmerich A., 2015 Influence of low-level prenatal exposure to PCDD/Fs and PCBs on empathizing, systemizing and autistic traits: results from the Duisburg birth cohort study. PLoS One 10: e0129906. 26066795 10.1371/journal.pone.0129906 PMC4466566

[bib55] Ota S. Horigome K. Ishii T. Nakai M. Hayashi K., 2009 Metformin suppresses glucose-6-phosphatase expression by a complex I inhibition and AMPK activation-independent mechanism. Biochem. Biophys. Res. Commun. 388: 311–316. 19664596 10.1016/j.bbrc.2009.07.164

[bib56] Owen M. R. Doran E. Halestrap A. P., 2000 Evidence that metformin exerts its anti-diabetic effects through inhibition of complex 1 of the mitochondrial respiratory chain. Biochem. J. 348(Pt. 3): 607–614. 10839993 PMC1221104

[bib57] Pe’er I. Yelensky R. Altshuler D. Daly M. J., 2008 Estimation of the multiple testing burden for genomewide association studies of nearly all common variants. Genet. Epidemiol. 32: 381–385. 18348202 10.1002/gepi.20303

[bib58] Penell J. Lind L. Fall T. Syvänen A.-C. Axelsson T., 2014 Genetic variation in the CYP2B6 gene is related to circulating 2,2′,4,4′-tetrabromodiphenyl ether (BDE-47) concentrations: an observational population-based study. Environ. Health 13: 34. 24885815 10.1186/1476-069X-13-34 PMC4024654

[bib59] Petersen M. S. Halling J. Bech S. Wermuth L. Weihe P., 2008 Impact of dietary exposure to food contaminants on the risk of Parkinson’s disease. Neurotoxicology 29: 584–590. 18455239 10.1016/j.neuro.2008.03.001

[bib60] Petry C. J. Ong K. K. Dunger D. B., 2007 Does the fetal genotype affect maternal physiology during pregnancy? Trends Mol. Med. 13: 414–421. 17900986 10.1016/j.molmed.2007.07.007

[bib61] Petry C. J. Beardsall K. Dunger D. B., 2014 The potential impact of the fetal genotype on maternal blood pressure during pregnancy. J. Hypertens. 32: 1553–1561, discussion 1561. 24842698 10.1097/HJH.0000000000000212

[bib62] Pruim R. J. Welch R. P. Sanna S. Teslovich T. M. Chines P. S., 2011 LocusZoom: regional visualization of genome-wide association scan results. Bioinformatics 27: 2336–2337. 10.1093/bioinformatics/btq419 PMC2935401 20634204

[bib63] Purcell S. Neale B. Todd-Brown K. Thomas L. Ferreira M. A. R., 2007 PLINK: a tool set for whole-genome association and population-based linkage analyses. Am. J. Hum. Genet. 81: 559–575. 17701901 10.1086/519795 PMC1950838

[bib64] R Core Team, 2014 R: A language and environment for statistical computing. R Foundation for Statistical Computing, Vienna, Austria. Available at: http://www.R-project.org/

[bib65] Rice D. Barone S., 2000 Critical periods of vulnerability for the developing nervous system: evidence from humans and animal models. Environ. Health Perspect. 108: 511–533. 10852851 10.1289/ehp.00108s3511 PMC1637807

[bib66] Risch N. Merikangas K., 1996 The future of genetic studies of complex human diseases. Science 273: 1516–1517. 8801636 10.1126/science.273.5281.1516

[bib67] Rogan W. J. Chen A., 2005 Health risks and benefits of bis(4-chlorophenyl)-1,1,1-trichloroethane (DDT). Lancet 366: 763–773. 16125595 10.1016/S0140-6736(05)67182-6

[bib68] Salihovic S. Lampa E. Lindström G. Lind L. Lind P. M., 2012 Circulating levels of persistent organic pollutants (POPs) among elderly men and women from Sweden: results from the Prospective Investigation of the Vasculature in Uppsala Seniors (PIVUS). Environ. Int. 44: 59–67. 22361238 10.1016/j.envint.2012.01.011

[bib69] Sandin S. Lichtenstein P. Kuja-Halkola R. Larsson H. Hultman C. M., 2014 The familial risk of autism. JAMA 311: 1770–1777. 24794370 10.1001/jama.2014.4144 PMC4381277

[bib70] Settembre C. Annunziata I. Spampanato C. Zarcone D. Cobellis G., 2007 Systemic inflammation and neurodegeneration in a mouse model of multiple sulfatase deficiency. Proc. Natl. Acad. Sci. USA 104: 4506–4511. 17360554 10.1073/pnas.0700382104 PMC1810506

[bib71] Shishikura M. Nakamura F. Yamashita N. Uetani N. Iwakura Y., 2016 Expression of receptor protein tyrosine phosphatase δ, PTPδ, in mouse central nervous system. Brain Res. 1642: 244–254. 27026654 10.1016/j.brainres.2016.03.030

[bib72] Sjödin A. McGahee E. E. Focant J. F. Jones R. S. Lapeza C. R., 2004 Semiautomated high-throughput extraction and cleanup method for the measurement of polybrominated diphenyl ethers and polybrominated and polychlorinated biphenyls in breast milk. Anal. Chem. 76: 4508–4514. 15283595 10.1021/ac0495384

[bib73] Sjödin A. Jones R. S. Caudill S. P. Wong L. Turner W. E., 2013 Polybrominated diphenyl ethers, polychlorinated biphenyls, and persistent pesticides in serum from the National Health and Nutrition Examination Survey: 2003–2008. Environ. Sci. Technol. 48: 753–760. 24298999 10.1021/es4037836 PMC4755520

[bib74] Tsang K. M. Croen L. A. Torres A. R. Kharrazi M. Delorenze G. N., 2013 A genome-wide survey of transgenerational genetic effects in autism. PLoS One 8: e76978. 24204716 10.1371/journal.pone.0076978 PMC3811986

[bib75] Visscher P. M. Hemani G. Vinkhuyzen A. A. E. Chen G. B. Lee S. H., 2014 Statistical power to detect genetic (co)variance of complex traits using SNP data in unrelated samples. PLoS Genet. 10: e1004269. 24721987 10.1371/journal.pgen.1004269 PMC3983037

[bib76] Vizcaino E. Grimalt J. O. Fernández-Somoano A. Tardon A., 2014 Transport of persistent organic pollutants across the human placenta. Environ. Int. 65: 107–115. 24486968 10.1016/j.envint.2014.01.004

[bib77] Vuong A. M. Braun J. M. Sjödin A. Webster G. M. Yolton K., 2016 Prenatal polybrominated diphenyl ether exposure and body mass index in children up to 8 years of age. Environ. Health Perspect. 124: 1891–1897. 27285825 10.1289/EHP139 PMC5132628

[bib78] Windham G. C. Anderson M. C. Croen L. A. Smith K. S. Collins J., 2011 Birth prevalence of autism spectrum disorders in the San Francisco Bay area by demographic and ascertainment source characteristics. J. Autism Dev. Disord. 41: 1362–1372. 21264681 10.1007/s10803-010-1160-2

[bib79] Yang J. Benyamin B. McEvoy B. P. Gordon S. Henders A. K., 2010 Common {SNPs} explain a large proportion of the heritability for human height. Nat. Genet. 42: 565–569. 20562875 10.1038/ng.608 PMC3232052

[bib80] Yang J. Lee S. H. Goddard M. E. Visscher P. M., 2011 GCTA: a tool for genome-wide complex trait analysis. Am. J. Hum. Genet. 88: 76–82. 21167468 10.1016/j.ajhg.2010.11.011 PMC3014363

[bib81] Zhao Y. Ruan X. Li Y. Yan M. Qin Z., 2013 Polybrominated diphenyl ethers (PBDEs) in aborted human fetuses and placental transfer during the first trimester of pregnancy. Environ. Sci. Technol. 47: 5939–5946. 23621775 10.1021/es305349x

