# Supplementary material for: Independent Maternal and Fetal Genetic Effects on Midgestational Circulating Levels of Environmental Pollutants
Source: G3 (Bethesda). 2017 Feb 24;7(4):1287–99. doi: 10.1534/g3.117.039784 (PMC5386877; doi:10.1534/g3.117.039784)
Supplement: Supplementary file 1 [file 1287FileS1.pdf]

Table S1. Self-reported ancestry of n=790 mothers

Ancestry group	N	percentage (%)	N cases	N controls	Stratification[a]
HIS	329	41.6%	148	181	PC1****; PC2***; PC5**
CAU	273	34.5%	141	132	NS
ASN	122	15.4%	68	54	PC9*
SOUTH ASN	23	2.9%	10	13	NS
AFR	21	2.7%	11	10	PC8*
OTHERS	22	2.8%	10	12	

[a] Significant differences across ancestry components between case-control data tested with two-sample Mann-Whitney Wilcoxon test; ****p-value < 0.0005; *** P<0.001; ** P<0.01; *P<0.05; . P<0.1

Abbreviations: CAU: Caucasians; ASN: Asians; HIS: Hispanics; SOUTH ASN: Indians; AFR: African-Americans; OTHERS: mixed ancestry, unknown ancestry, other ancestry (based on self-reported ancestry groups); PC: ancestry components

Table S2. Significance of sociodemographic confounding factors in linear regression models of 19 organohalogens and PCB and PBDE sums

	age		multiparity		education		country of birth		month of birth		offspring gender		ancestry
**compound**	*dir*	*P*	*dir*	*P*	*dir*	*P*	*dir*	*P*	*dir*	*P*	*dir*	*P*	*P*
Persistent Pesticides													
p,p'-DDE	+	***	-	***	-	**	+	***	+	**	+	.	***
T-NONA	+	***	-	***	-	NS	-	***	-	NS	-	NS	**
Polybrominated diphenyl ethers and biphenyls													
BB-153	+	***	+	NS	+	***	-	***	+	**	-	NS	**
BDE-28	-	*	+	*	-	NS	-	.	-	*	+	NS	NS
BDE-47	-	***	+	*	+	NS	-	**	-	NS	+	NS	NS
BDE-99	-	***	+	***	-	NS	-	.	-	NS	+	NS	*
BDE-100	-	***	+	*	+	.	-	**	-	.	+	NS	NS
BDE-153	-	**	+	NS	-	.	-	***	-	.	-	NS	***
Sum PBDE	-	**	+	**	+	.	-	**	-	NS	+	NS	*
Polyclorinated biphenyls													
PCB28	+	NS	-	NS	-	*	+	NS	-	**	+	*	*
PCB99	+	***	-	***	+	**	-	.	+	NS	-	NS	***
PCB118	+	***	-	***	+	*	-	*	-	NS	+	NS	***
PCB153	+	***	-	***	+	***	+	NS	+	.	-	NS	***
PCB170	+	***	-	**	+	**	+	NS	+	NS	-	NS	***
PCB180	+	***	-	*	+	***	+	NS	+	NS	-	NS	***
PCB187	+	***	-	NS	+	***	+	NS	+	NS	-	NS	***
PCB194	+	***	-	NS	+	*	-	NS	+	*	-	NS	***
PCB199	+	***	-	NS	+	**	-	*	+	NS	-	NS	***
PCB138/158	+	***	-	***	+	**	+	NS	+	NS	-	NS	***
PCB196/203	+	***	-	NS	+	**	-	**	+	NS	-	NS	***
Sum PCB	+	***	-	.	+	.	+	NS	+	NS	+	NS	***

Abbreviations: *dir*: direction; *P*: p-value; NS: not significant
****p-value < 0.0005; *** P<0.001; ** P<0.01; *P<0.05; . P<0.1

Table S3. Compressed maternal genome-wide summary-level results for each compound

Table S4. Compressed neonatal genome-wide summary-level results for each compound

Table S5. Quartile analysis in EMA genotyped dataset consistent with EMA full dataset showed in Table 3 in Lyall et al., 2016(Lyall *et al.*2016).

	**Odds of ASD relative to GP controls**		
	**Unadjusted OR (95% CI)**	**AOR (95% CI)^**	**AOR (95% CI)#**
PCB138_158			
Q1	1	1	1
Q2	0.97 (0.91, 1.03)	0.99 (0.93, 1.06)	1.00 (0.94, 1.06)
Q3	0.99 (0.93, 1.05)	0.98 (0.92, 1.04)	0.97 (0.92, 1.04)
Q4	**1.14 (1.06, 1.21)**	1.04 (0.99, 1.10)	1.03 (0.98,1.09)
PCB153			
Q1	1	1	1
Q2	0.95 (0.89, 1.01)	0.97 (0.91, 1.03)	0.98 (0.92, 1.03)
Q3	0.97 (0.91, 1.03)	0.96 (0.90, 1.03)	0.96 (0.90, 1.02)
Q4	**1.17 (1.09, 1.25)**	**1.07 (1.02, 1.13)**	**1.06 (1.01, 1.12)**

Abbreviations: ASD Autism spectrum disorder; GP general population controls. Bold indicates statistical significance (p<0.05)

^Adjusted for matching factors (offspring gender, month and year of birth), maternal age (continuous), maternal race/ethnicity (Non-Hispanic White, Asian, Black/Pacific Islander/or other, Hispanic, or missing), maternal weight at time of sample collection (quartiles), parity (multi- vs primiparous), and maternal education (<high school, high school, college, graduate). Removal of race/ethnicity from these models did not materially alter estimates; maternal age and parity were the only significant covariates in models and adjustment for these factors only yielded AORs similar to fully adjusted estimates. Models adjusted only for matching factors yielded estimates nearly identical as unadjusted models.

#Adjusted for EMA genotyped dataset covariates (offspring gender, month of birth, maternal age, maternal education, country of birth, multiparity, PC1-10)

Table S6. Maternal top genetic loci genome-wide and suggestively associated with mid-gestational levels of organohalogens

compound	SNP	CHR	BP	MAF	beta	SE	P	locus
Persistent Pesticides								
**p,p'-DDE**	**rs7259965**	**19**	**41521532**	**0.29**	**0.32**	**0.05**	**$7.8x10^{-11}$**	***CYP2B6***
T-NONA	rs1181906	7	155977316	0.14	-0.17	0.04	$5.7\ x10^{-6}$	*lincRNA*
Polybrominated diphenyl ethers and biphenyls								
BB-153	*rs56385737*	*18*	*4414795*	*0.05*	*0.44*	*0.08*	$3.1x10^{-7}$	*DLGAP1*
**BDE-28**	**rs11789653**	**9**	**17993589**	**0.03**	**0.65**	**0.12**	**$3.2x10^{-8}$**	***ADAMTSL1 SH3GL2***
BDE-47	*rs2855812*	*6*	*31472720*	*0.21*	*-0.33*	*0.07*	$7.9x10^{-7}$	*MICB*
**BDE-99**	**rs7259965**	**19**	**41521532**	**0.29**	**0.32**	**0.06**	**$2.1x10^{-8}$**	***CYP2B6***
BDE-100	*rs2855812*	*6*	*31472720*	*0.21*	*-0.35*	*0.07*	$2.9x10^{-7}$	*MICB*
BDE-153	rs17506613	8	38594656	0.48	-0.27	0.06	$1.1x10^{-6}$	*TACC1*
Sum PBDE	rs2855812	6	31472720	0.21	-0.29	0.06	$1.8x10^{-6}$	*MICB*
Polyclorinated biphenyls								
PCB28	rs11727994	4	91911417	0.35	-0.34	0.07	$1.6x10^{-6}$	*TMSB4XP8*
PCB99	rs1812081	2	134279837	0.40	0.13	0.03	$4.8x10^{-6}$	*MGAT5*
PCB118	rs8076755	17	13788655	0.02	-0.51	0.11	$7.5x10^{-6}$	*HS3ST3A1 COX10*
PCB153	rs76546227	15	33276215	0.02	-0.55	0.11	$1.0x10^{-6}$	*FMN1*
PCB170	rs10826182	10	60164820	0.32	0.15	0.03	$2.1x10^{-6}$	*TFAM BICC1*
PCB180	rs4946957	6	109641411	0.39	-0.15	0.03	$1.2x10^{-6}$	*CCDC162P CEP57L1 CD164*
PCB187	*rs10500669*	*11*	*6328472*	*0.23*	*0.20*	*0.04*	$2.5x10^{-7}$	*PRKCDBP*
PCB194	*rs75107653*	*3*	*4181811*	*0.02*	*0.57*	*0.11*	$1.3x10^{-7}$	*SETMAR SUMF1*
PCB199	*rs4945829*	*6*	*109638828*	*0.43*	*-0.17*	*0.03*	$3.7x10^{-7}$	*CCDC162P CEP57L1*

								CD164
PCB138/158	rs76546227	15	33276215	0.02	-0.59	0.12	$9.4x10^{-7}$	*FMN1*
PCB196/203	*rs75107653*	*3*	*4181811*	*0.02*	*0.57*	*0.11*	$8.6x10^{-8}$	*SETMAR*<br>*SUMF1*
PCB196/203	*rs4945829*	*6*	*109638828*	*0.43*	*-0.16*	*0.03*	$1.1x10^{-7}$	*CCDC162P*<br>*CEP57L1*<br>*CD164*
Sum PCB	*rs35560597*	*5*	*52678868*	*0.01*	*0.79*	*0.15*	$7.9x10^{-8}$	*FST*<br>*NDUFS4*

In each category the results are shown in alphanumeric order. The genome-wide significant associations are shown in **bold** and the suggestively significant associations are in *italics*. Genome-wide association threshold is P=$5x10^{-8}$ and suggestive association threshold is P=$5x10^{-7}$.
Abbreviations: CHR: chromosome; BP: position: SE: standard error

Table S7. Suggestively and nominally significant association between maternal chr3:rs75107653, chr6:rs4945829, chr11:rs10500669 and PCB congeners

	chr3:rs75107653 - MAF=0.016				chr6:rs4945829 - MAF=0.43				chr11:rs10500669 - MAF=0.23			
compound	N	beta	SE	p-value	N	beta	SE	p-value	N	beta	SE	p-value
PCB28	763	0.5	0.24	3.7x10^{-2}	762	0.05	0.07	NS	760	-0.05	0.08	NS
PCB118	714	0.24	0.11	2.7x10^{-2}	713	-0.07	0.03	2.4x10^{-2}	711	0.04	0.03	NS
PCB153	755	0.24	0.11	2.4x10^{-2}	754	-0.12	0.03	2.8x10^{-5}	752	0.12	0.03	4.1x10^{-4}
PCB170	727	0.39	0.11	1.9x10^{-4}	726	-0.13	0.03	1.2x10^{-5}	724	0.15	0.03	9.5x10^{-6}
PCB180	759	0.41	0.11	1.9x10^{-4}	758	-0.14	0.03	3.6x10^{-6}	756	0.15	0.03	1.7x10^{-5}
PCB187	714	0.38	0.12	1.6x10^{-3}	713	-0.16	0.03	2.6x10^{-6}	*711*	*0.2*	*0.04*	*2.5x10^{-7}*
PCB194	*712*	*0.57*	*0.11*	*1.3x10^{-7}*	711	-0.15	0.03	6.5x10^{-7}	709	0.15	0.03	1.5x10^{-6}
PCB199	715	0.56	0.12	1.6x10^{-6}	*714*	*-0.17*	*0.03*	*3.7x10^{-7}*	712	0.17	0.04	5.2x10^{-6}
PCB138/158	752	0.18	0.11	NS	751	-0.12	0.03	9.1x10^{-5}	749	0.11	0.04	3.1x10^{-3}
PCB196/203	*715*	*0.57*	*0.11*	*8.6x10^{-8}*	*714*	*-0.16*	*0.03*	*1.1x10^{-7}*	712	0.17	0.03	1.0x10^{-6}
Sum PCB	706	0.4	0.12	4.8x10^{-4}	705	-0.04	0.03	NS	703	0.07	0.04	NS

In each category the results are shown in alphanumeric order. Suggestively significant associations are shown in *italics*. Suggestive association threshold is P=5x10^{-7}. Abbreviations: MAF: minor allele frequency; SE: standard error; NS: not significant

Table S8. Genome wide and nominally significant association in fetal chr9:rs72692916 and chr18:rs72913475 across organohalogens

	chr9:rs72692916 - MAF=0.05				chr18:rs72913475 - MAF=0.08			
compound	N	beta	SE	p-value	N	beta	SE	p-value
Persistent Pesticides								
T-NONA	644	0.05	0.07	NS	664	0.15	0.05	$5.5x10^{-3}$
Polybrominated diphenyl ethers and biphenyls								
BB-153	635	-0.15	0.09	NS	655	0.16	0.07	$2.3x10^{-2}$
BDE-28	629	-0.10	0.08	NS	649	0.26	0.07	$1.2x10^{-4}$
BDE-47	686	-0.20	0.12	NS	*708*	*0.51*	*0.10*	*$2.9x10^{-7}$*
BDE-99	666	-0.16	0.12	NS	688	0.33	0.10	$7.2x10^{-4}$
BDE-100	675	-0.27	0.13	$3.6x10^{-2}$	**697**	**0.55**	**0.10**	**$4.6x10^{-8}$**
BDE153	674	-0.29	0.12	$2.0x10^{-2}$	696	0.44	0.10	$7.1x10^{-6}$
Sum PBDE	640	-0.26	0.11	$1.8x10^{-2}$	660	0.35	0.09	$1.8x10^{-4}$
Polyclorinated biphenyls								
PCB99	642	-0.22	0.06	$3.0x10^{-4}$	709	0.10	0.05	$4.3x10^{-2}$
PCB118	644	-0.20	0.07	$2.9x10^{-3}$	664	0.09	0.06	NS
PCB153	681	-0.30	0.07	$1.0x10^{-5}$	703	0.13	0.05	$2.0x10^{-2}$
PCB170	654	-0.32	0.07	$1.7x10^{-6}$	675	0.12	0.55	$2.9x10^{-2}$
PCB180	683	-0.34	0.07	$7.6x10^{-7}$	705	0.12	0.55	$3.6x10^{-2}$
PCB187	**644**	**-0.42**	**0.08**	**$2.8x10^{-8}$**	664	0.11	0.06	NS
PCB194	642	-0.31	0.07	$7.0x10^{-6}$	662	0.10	0.06	NS
PCB199	645	-0.36	0.07	$1.3x10^{-6}$	665	0.09	0.06	NS
PCB138/158	676	-0.26	0.07	$2.8x10^{-4}$	698	0.11	0.06	NS
PCB196/203	645	-0.33	0.07	$1.5x10^{-6}$	665	0.12	0.05	$3.5x10^{-2}$
Sum PCB	637	-0.26	0.07	$4.9x10^{-4}$	657	0.08	0.06	NS

In each category the results are shown in alphanumeric order. The genome-wide significant associations are shown in **bold** and the suggestively significant associations are in *italics*. Genome-wide association threshold is P=$5x10^{-8}$ and suggestive association threshold is P=$5x10^{-7}$. Abbreviations: MAF: minor allele frequency; SE: standard error; NS: not significant

Table S9. Fetal genetic loci genome-wide, suggestively and nominally associated with mid-gestational levels of organohalogens

compound	SNP	CHR	BP	MAF	beta	SE	p-value	locus
PCB199	rs1400087	1	106,108,925	0.07	0.33	0.07	$7.6x10^{-7}$	lincRNA
PCB180	*rs1400087*	*1*	*106,108,925*	*0.08*	*0.32*	*0.06*	*$1.1x10^{-7}$*	lincRNA
PCB199	rs6660284	1	106,115,714	0.08	0.34	0.07	$7.2x10^{-7}$	lincRNA
PCB180	*rs6660284*	*1*	*106,115,714*	*0.08*	*0.33*	*0.06*	*$1.2x10^{-7}$*	lincRNA
BDE-100	*rs829661*	*2*	*30,726,691*	*0.19*	*0.37*	*0.07*	*$2.3x10^{-7}$*	*LCLAT1*
BB-153	*rs114147886*	*3*	*17,854,598*	*0.02*	*0.82*	*0.15*	*$7.6x10^{-8}$*	near *TBC1D5*
BDE-28	rs76882575	3	179,782,262	0.04	0.49	0.10	$8.1x10^{-7}$	near *PEX5L*
PCB118	*rs11755413*	*6*	*125,846,604*	*0.10*	*0.28*	*0.05*	*$2.8x10^{-7}$*	between *HEY2* and *HDDC2*
BDE-47	rs1034591	7	114,443,038	0.43	-0.27	0.05	$6.3x10^{-7}$	between *FOXP2* and *MDFIC*
**PCB187**	**rs72692916**	**9**	**8,446,518**	**0.05**	**-0.42**	**0.08**	**$2.8x10^{-8}$**	***PTPRD***
BDE-28	rs72896141	11	5,845,481	0.05	0.40	0.08	$5.6x10^{-7}$	*TRIM5*
BB-153	*rs763311*	*12*	*12,796,067*	*0.01*	*0.89*	*0.17*	*$1.6x10^{-7}$*	*CREBL2*
PCB138/158	*rs61989804*	*14*	*65,697,140*	*0.05*	*-0.38*	*0.07*	*$1.3x10^{-7}$*	pseudogenes
BDE-153	*rs8054028*	*16*	*73,159,970*	*0.17*	*-0.39*	*0.07*	*$2.1x10^{-7}$*	near c16orf47
BDE-99	rs117896058	18	8,168,060	0.01	1.33	0.26	$5.4x10^{-7}$	*PTPRM*
**BDE-100**	**rs72913475**	**18**	**44,170,156**	**0.08**	**0.55**	**0.10**	**$4.6x10^{-8}$**	***LOXHD1***

In each category the results are shown in alphanumeric order. The genome-wide significant associations are shown in **bold** and the suggestively significant associations are in *italics*. Genome-wide association threshold is P=$5x10^{-8}$ and suggestive association threshold is P=$5x10^{-7}$. Abbreviations: CHR: chromosome; BP: position: SE: standard erro

Figure Legends

Figure S1. Multi-dimensional scaling plot of EMA (a) maternal and (b) newborn cohort, and (c) non-overlapping Caucasian, Asian and Hispanic EMA maternal groups. Each individual is represented with a dot and the distance between two individuals represents the genetic distance between them and summarizes the ancestry differences. The X-axis represents the first ancestry coordinate that explains 57.6% and 52.7% of the total genetic variance of the (a) maternal and (b) fetal cohort, respectively. The Y-axis represents the second ancestry coordinate that explains 25.3% and 25.5% of the total genetic variance of the (a) maternal and (b) fetal cohort, respectively; (c) Clustering of 90% of EMA mothers in three non-overlapping ethnic groups. Abbreviations: White: Caucasians; ASN: Asians; HIS: Hispanics; SOUTH ASN: Indians; AFR: African-Americans; OTHERS: mixed ancestry, unknown ancestry, other ancestry (based on self-reported ancestry groups); not compared: individuals not used in analyses comparing ancestry groups.

Figure S2. Residual PBDE congener serum levels by ASD outcome after covariate adjustment.

Boxplot of residual levels of BDE-100 (a), BDE-153 (b) and Sum PBDE (c) in mothers of ASD controls (white) and ASD cases (light grey) obtained with a linear regression after adjustment for sociodemographic covariates. Two-sample Mann-Whitney Wilcoxon test: * $P < 0.05$; ** $P < 0.01$.

Figure S3. Linear non-parametric correlation coefficients for 19 organohalogens. Correlation coefficients across organhalogens (total sum of PBDEs and PCBs were excluded) calculated with Spearman's test. From weakest to strongest, positive correlation coefficients are represented in a red gradient; negative correlation coefficients are displayed in a blue gradient.

Figure S4. Linkage disequilibrium regional genomic plots of maternal suggestively associated SNPs with PCBs and BB-153 serum levels. (a) rs35560597 associated with sum PCB maps near *FST* and *NDUFS4* on chromosome 5q11.2 (β=0.79, SE=0.15, P=7.9x10^{-8}), (b) rs75107653 on chromosome 3p26.1 (PCB196/203, β=0.57, SE=0.11, P=8.6x10^{-8}) maps near *SETMAR* and *SUMF1*, (c) rs4945829 on chromosome 6q21 (PCB196/203, β=-0.16, SE=0.03, P=1.1x10^{-7}) maps to *CCDC162P* and near *CD164*, (d) rs10500669 on chromosome 11p15.4 (PCB187, β=0.20, SE=0.04, P=2.5x10^{-7}) is located near *PRKCDBP* and (e) rs56385737 on chromosome18p11.31 (BB-153, beta=0.44, SE=0.08, P=3.1x10^{-7}) maps to the *DLGAP1* gene The X-axis represents the genomic position; the Y-axis shows the negative logarithm of the observed association p-value for each tested SNP and the recombination rate. Plotted with Locuzoom tool(Pruim *et al.*2011;).

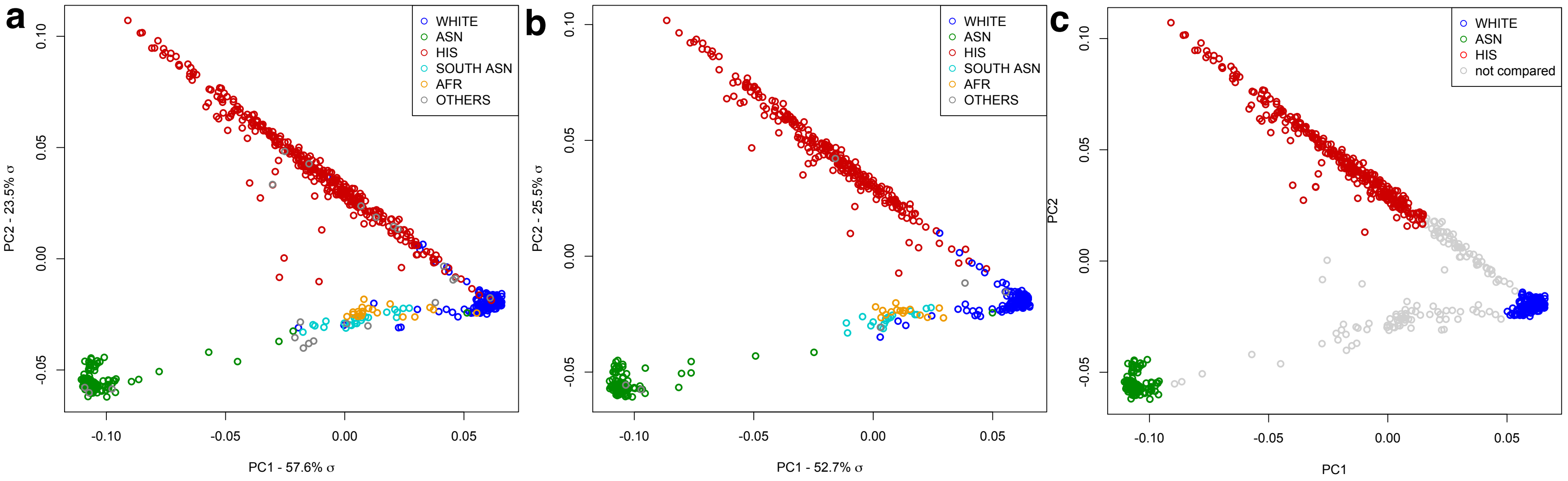
a
b
c
WHITE
ASN
HIS
SOUTH ASN
AFR
OTHERS
not compared
PC1 - 57.6% σ
PC2 - 23.5% σ
PC1 - 52.7% σ
PC2 - 25.5% σ
PC1
PC2
-0.10
-0.05
0.00
0.05
0.10

Figure S1

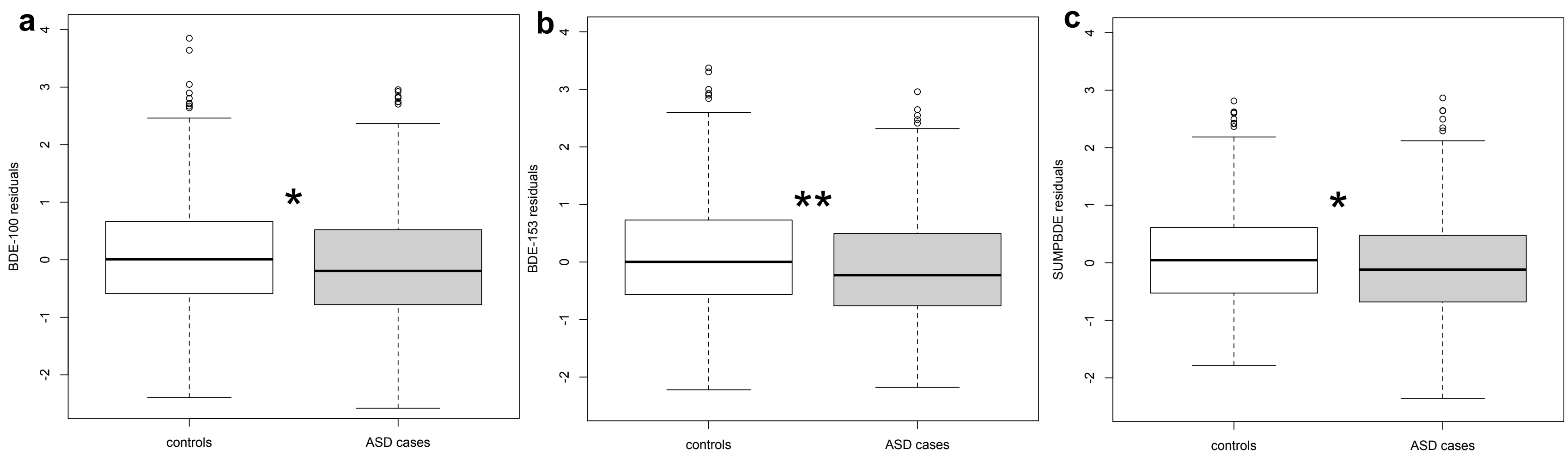
a
b
c
BDE-100 residuals
BDE-153 residuals
SUMPBDE residuals
*
**
*
controls
ASD cases
4
3
2
1
0
-1
-2

Figure S2

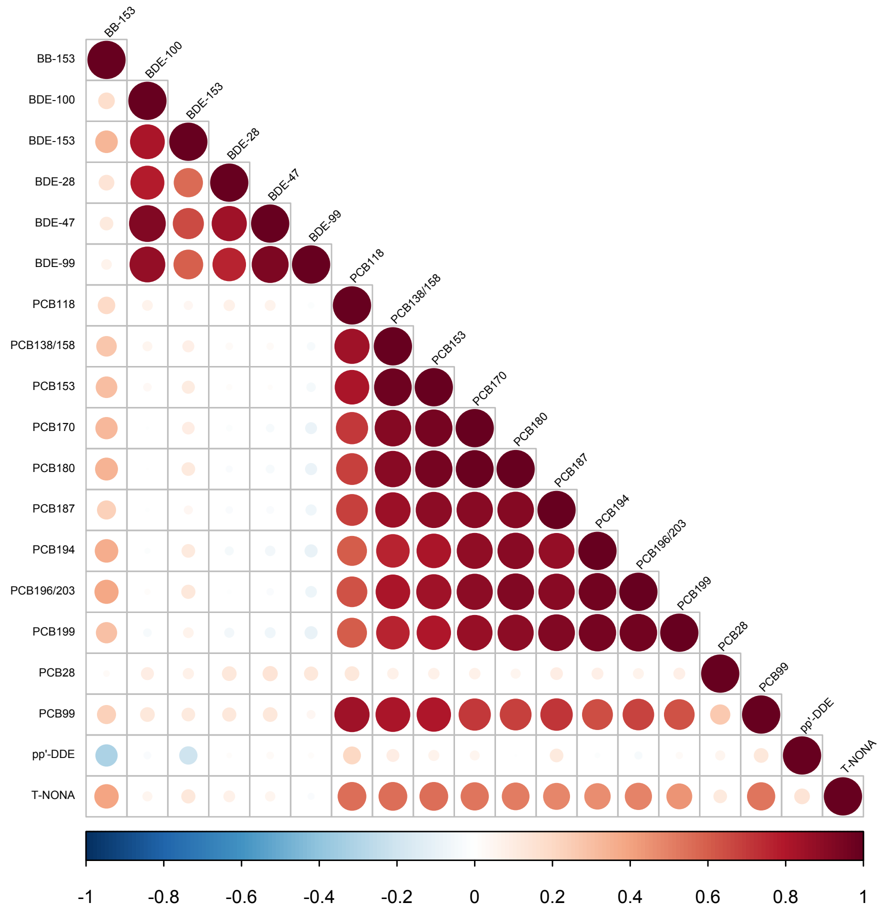

BB-153
BDE-100
BDE-153
BDE-28
BDE-47
BDE-99
PCB118
PCB138/158
PCB153
PCB170
PCB180
PCB187
PCB194
PCB196/203
PCB199
PCB28
PCB99
pp'-DDE
T-NONA
-1
-0.8
-0.6
-0.4
-0.2
0
0.2
0.4
0.6
0.8
1

Figure S3

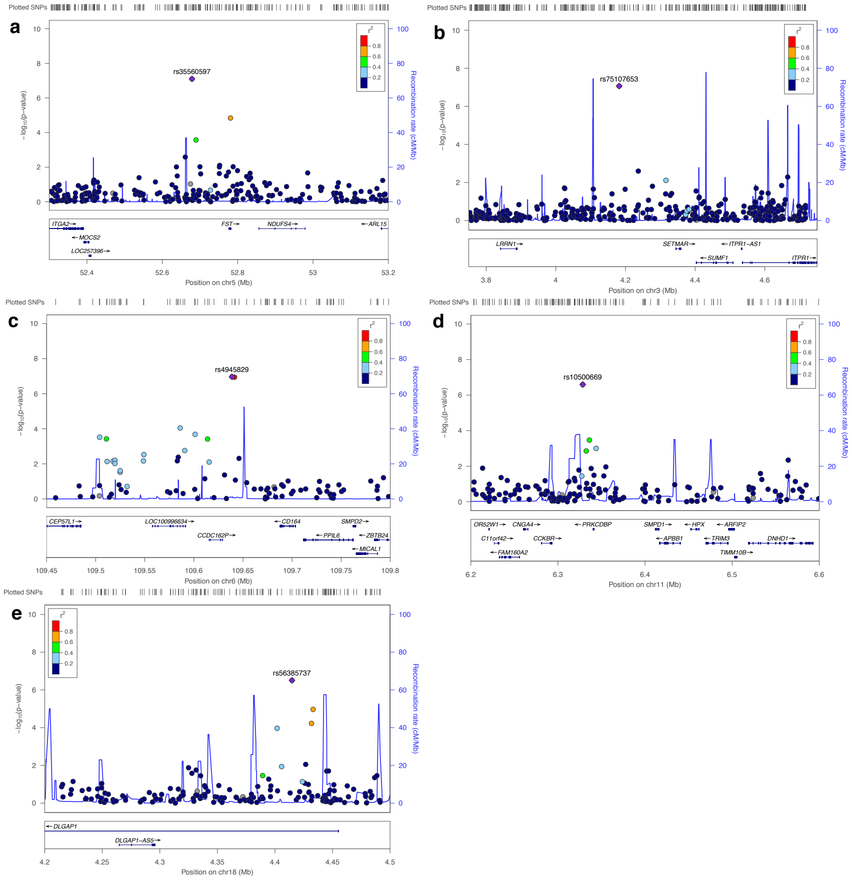
a
rs35560597
ITGA2
MOCS2
LOC257396
FST
NDUFS4
ARL15
Position on chr5 (Mb)
b
rs75107653
LRRN1
SETMAR
ITPR1-AS1
SUMF1
ITPR1
Position on chr3 (Mb)
c
rs4945829
CEP57L1
LOC100996634
CCDC162P
CD164
SMPD2
PPIL6
ZBTB24
MICAL1
Position on chr6 (Mb)
d
rs10500669
OR52W1
CNGA4
PRKCDBP
SMPD1
HPX
ARFIP2
C11orf42
CCKBR
APBB1
TRIM3
DNHD1
FAM160A2
TIMM10B
Position on chr11 (Mb)
e
rs56385737
DLGAP1
DLGAP1-AS5
Position on chr18 (Mb)
Plotted SNPs
-log10(p-value)
Recombination rate (cM/Mb)
r²

Figure S4
